# Structural basis for dual mechanism of Cas2/3 nuclease inhibition by anti-CRISPR protein AcrIF19

**DOI:** 10.1038/s41467-026-73156-3

**Published:** 2026-05-19

**Authors:** Yuanshuo Sa, Chunlei Liu, Lingguang Yang, Ling Yue, Limin Zhu, Ying Guo, Ruimei Wang, Yafei Wang, Yue Feng, Yong Wang, Yi Zhang, Wenhe Wang, Yongchao Xie

**Affiliations:** 1 National Key Laboratory of Wheat Improvement, College of Life Sciences, Shandong Agricultural University, Tai’an, China; 2 Crop Microstructure Engineering Research Center of Shandong Province, Shandong Agricultural University, Tai’an, China; 3 School of Pharmacy, Yichun University, Yichun, China; 4 State Key Laboratory of Green Biomanufacturing, College of Life Science and Technology, Beijing University of Chemical Technology, Beijing, China; 5 Department of Biological Science and Engineering, School of Chemistry and Biological Engineering, University of Science and Technology Beijing, Beijing, China

**Keywords:** Electron microscopy, Molecular conformation, Bacteriophages, CRISPR-Cas systems, Enzyme mechanisms

## Abstract

CRISPR-Cas systems are prokaryotic immune mechanisms often targeted by phage-encoded anti-CRISPR (Acr) proteins. This study characterizes AcrIF19, a potent inhibitor of the type I-F system in *Pectobacterium atrosepticum*. The cryo-EM structure of the apo Cas2/3 and Cas2/3-AcrIF19 complex reveals a dual inhibitory mechanism. AcrIF19 employs a negatively charged β_2_-β_3_ loop to sterically occlude the non-target DNA strand entry channel, acting as a competitive inhibitor to disrupt Cas2/3 recruitment. Concurrently, this steric occlusion impedes ssDNA-mediated allosteric activation, which locks the critical helix-like loop motif in an inhibitory conformation and thereby abrogates DNA cleavage activity. AcrIF19 represents an anti-CRISPR protein inhibiting Cas2/3 via two different mechanisms, integrating a competitive ssDNA inhibitor with an allosteric blockade to suppress both target recruitment and DNA cleavage.

## Abstract (web summary)

CRISPR-Cas systems are prokaryotic immune defenses commonly targeted by phage-encoded anti-CRISPR (Acr) proteins. Here, authors report cryo-EM structures of Cas2/3 and its complex with AcrIF19 from *Pectobacterium atrosepticum*, and uncover its dual inhibitory mechanism against Cas2/3 function.

## Introduction

In prokaryotes, CRISPR-Cas systems serve as adaptive immune defenses against mobile genetic elements, such as phages^1–3^. Found in approximately 40% of bacteria and 85% of archaea^4,5^, these systems are classified into two classes: Class 1 (types I, III, IV) relies on multi-subunit complexes, while Class 2 (types II, V, VI) employs a single effector protein^6^. Immune responses involve three stages: integrating invader DNA (adaptation), producing crRNA-guided complexes (expression), and targeted cleavage of foreign nucleic acids (interference)^7,8^. The Type I-F subtype contains Cas1, a Cas2/3 fusion protein, Cas8f (formerly Csy1), Cas5f (formerly Csy2), Cas7f (formerly Csy3), and Cas6f (formerly Csy4). During the interference phase, the Cas8f subunit of the Csy complex recognizes the PAM motif in invader DNA (e.g., phage-derived) and binds selectively to the target strand guided by crRNA, forming an R-loop structure without binding to the nontarget strand^9,10^. R-loop formation elongates the Csy complex and induces Cas8f-mediated 180° rotation of the Cas2/3-recruiting helical bundle, exposing its binding site to recruit Cas2/3. This bifunctional nuclease-helicase subsequently degrades dsDNA^11–15^.

As the core effector of type I CRISPR-Cas systems, Cas3 (or Cas2/3 in type I-F) possesses a 3'→5' ssDNA nuclease activity within its HD domain that is central to its function^16^. This activity drives both foreign DNA degradation and immune adaptation. During dsDNA targeting, Cascade/Csy recognizes PAMs and forms an R-loop to recruit Cas3. Cas3’s SF2 helicase then unwinds dsDNA, exposing ssDNA substrates that are subsequently cleaved by the HD domain via a processive “unwinding-cleavage” cycle. Progressive unwinding coupled with sequential hydrolysis of exposed strands ultimately fragments foreign DNA^17–19^. Subtype-specific variations further define Cas3 function: The type I-E system exhibits cis-cleavage (triggered by complete R-loop formation) and trans-cleavage (induced by partial target binding); type I-C achieves ATP-independent cleavage via Ni^2+^/Co^2+^ activation and structural rearrangements, including anchor loop flip toward Cas8 and RecA1 gate loop displacement from the HD active center; while type I-F Cas2/3 enables bidirectional degradation with preferential non-complementary strand cleavage^17–20^. At the regulatory level, Cas1 inhibits type I-F Cas2/3 through complex formation, whereas target-bound Cascade/Csy activates the nuclease. These collective insights elucidate Cas3’s structural mechanisms and subtype differentiation while providing key support for CRISPR-Cas3 genome editing optimization^19,21^.

Bacteriophages evade CRISPR-Cas systems via anti-CRISPR proteins^22^, first identified as five Acrs genes in *Pseudomonas aeruginosa*-infecting phages^17^. Over 100 Acr families now antagonize 12 CRISPR-Cas subtypes, including 27 Acrs targeting type I-F systems^23–28^. Structures and mechanisms are resolved for 18 Acrs (AcrIF1-11,13,14,23-27)^26,29^, revealing diverse interference-stage strategies. Notably, only AcrIF3 and AcrIF23 have been shown to suppress Cas2/3 recruitment or enzymatic activity through direct binding^30,31^. Specifically, AcrIF3 blocks Cas2/3 recruitment to the Csy-DNA complex via Cas8f domain mimicry, while AcrIF23 permits recruitment but inhibits Cas2/3’s enzymatic activity post-target hybridization^15,31,32^. Although AcrIF3’s inhibitory mechanism is relatively well established, the detailed basis for its recruitment inhibition remains incompletely understood, alongside the mechanism of AcrIF23’s cleavage-specific suppression; the functions of AcrIF12 and AcrIF15-22 also remain unclear.

Here, we determined the structure and mechanism of AcrIF19, an uncharacterized type I-F anti-CRISPR protein. Structures of monomeric auto-inhibited *P. atrosepticum* Cas2/3 and the Cas2/3-AcrIF19 complex reveal the enzyme’s conformational activation pathway. AcrIF19 enforces dual functional inhibition: its acidic β_2_-β_3_ loop sterically occludes the ssDNA entry channel, blocking substrate recruitment and preventing the allosteric transition required for RecA1 loop displacement and HD nuclease exposure. This locks Cas2/3 in its intrinsic auto-inhibited state, establishing a paradigm for CRISPR-Cas suppression that exploits the target enzyme’s conformational auto-regulation.

## Results

### AcrIF19 interacts with Cas2/3 and inhibits the CRISPR-Cas system in vitro

**Fig. 1:**
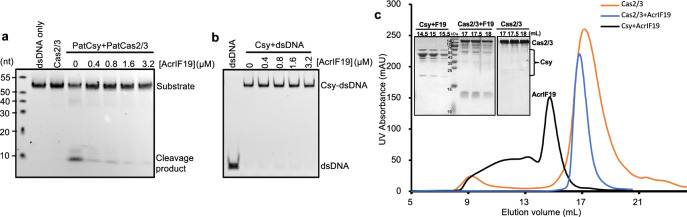
AcrIF19 interacts with Cas2/3 and inhibits the CRISPR-Cas system in vitro*.* **a** AcrIF19 inhibits the in vitro cleavage activity of the type I-F CRISPR-Cas system. Reactions were performed with 0.32 μM Csy complex, 0.64 μM Cas2/3, and 0.1 μM 54-bp dsDNA (5'-FAM in the non-target DNA strand, NTS). AcrIF19 was added with the indicated concentrations; **b** AcrIF19 does not inhibit the binding of Csy to dsDNA; **c** AcrIF19 binds to Cas2/3 not the Csy complex. For (**a–c**), the experiments were repeated independently three times, with similar results.

Previous studies have revealed that AcrIF19 inhibits the type I-F CRISPR-Cas system of *Pectobacterium atrosepticum* but not that of *P. aeruginosa*. To validate this species-specific inhibition, we expressed and purified the Csy complex and nuclease Cas2/3 from both *P. aeruginosa* and *P. atrosepticum*, respectively. Using in vitro DNA cleavage inhibition assays, we demonstrated that AcrIF19 specifically inhibits the immune activity of the type I-F CRISPR-Cas system in *P. atrosepticum* but not in *P. aeruginosa* (Fig. 1a and Supplementary Fig. 1a). To further elucidate the inhibitory mechanism of AcrIF19, we first performed electrophoretic mobility shift assays (EMSA) to investigate whether AcrIF19 interferes with Csy-mediated recognition and binding to double-stranded DNA (dsDNA; using the *P. atrosepticum* Csy complex). The results showed that AcrIF19 had no effect on this process, indicating that AcrIF19 may not exert its inhibitory function by targeting Csy (Fig. 1b). Given that AcrIF19 did not target Csy, we next used pull-down assays to test whether it interacts with nuclease Cas2/3. Our data showed that AcrIF19 binds directly to Cas2/3 (Supplementary Fig. 1b). To confirm that AcrIF19 inhibits the system by targeting Cas2/3 rather than the Csy complex, we co-expressed AcrIF19 with either the Csy complex or Cas2/3. Results indicated that AcrIF19 forms a stable complex with Cas2/3 but not with the Csy complex (Fig. 1c).

### Structural overview of Cas2/3-AcrIF19 complex

**Fig. 2:**
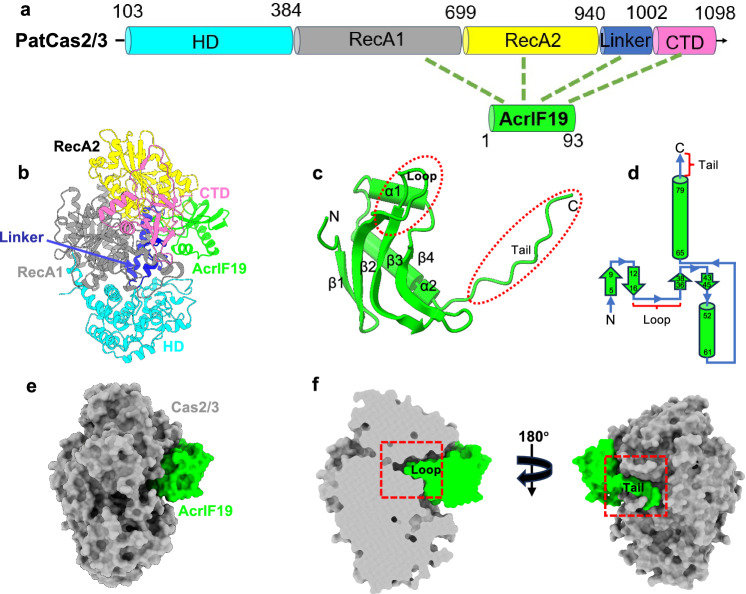
Overall cryo-EM structure of the Cas2/3-AcrIF19 complex. **a** A schematic of the domain architecture with different domains colored according to the key within the panel. Pre-existing interactions are indicated with green dashed lines; **b** Atomic structure of Cas2/3-AcrIF19 in cartoon representation. Domains and AcrIF19 are colored according to the key within the panel; **c** Secondary structure composition of AcrIF19; **d** Topology diagram of AcrIF19; **e** Surface representation of the Cas2/3-AcrIF19 complex. Components are colored according to the key within the panel; **f** Cross-sectional view of the Cas2/3-AcrIF19 complex. Two characteristic structural elements (loop and tail) of AcrIF19 are highlighted by a red dashed box.

To elucidate the inhibitory mechanism of AcrIF19, we determined the Cas2/3-AcrIF19 complex structure using single-particle cryo-electron microscopy (cryo-EM) at 2.97 Å resolution (Supplementary Fig. 2a, b and Supplementary Table 1). The atomic model of AcrIF19 was de novo constructed using cryo-EM map, and the Cas2/3 complex components were reconstructed. Initial analysis of Cas2/3 domains revealed that AcrIF19 interacts with the RecA1 (385-699), RecA2 (700-940), Linker (941-1002), and CTD (1003-1098) domains, but notably not with the HD domain (Fig. 2a, b). Further structural investigation showed that AcrIF19 adopts a fold comprising four antiparallel β-strands and two α-helices from N- to C-terminus, featuring a distinctive 10-amino acid C-terminal tail (Fig. 2c, d). Surface representation and cross-sectional analysis revealed that the loop connecting AcrIF19’s β_2_ and β_3_ strands inserts snugly into a concave binding groove on Cas2/3, facilitated by a highly matched interaction reminiscent of a canonical lock-and-key interaction. Concurrently, the C-terminal tail of AcrIF19 anchors tightly into a separate surface channel on Cas2/3 (Fig. 2e, f). Collectively, these results establish that AcrIF19 binds to Cas2/3 mainly through two structurally distinct functional units, the β_2_-β_3_ loop and the C-terminal tail, which are likely critical for its inhibitory mechanism.

### AcrIF19 mediates steric blockade of Cas2/3 ssDNA channel via its β_2_-β_3_ loop

**Fig. 3:**
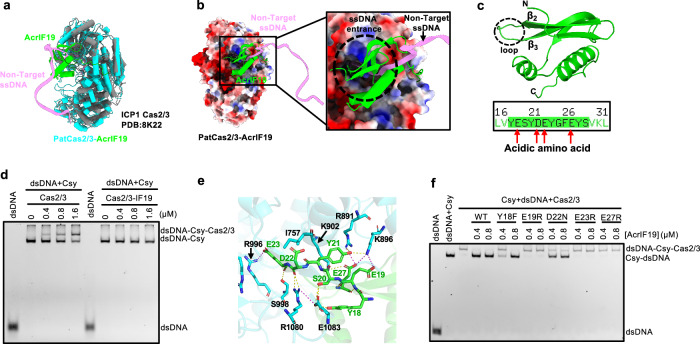
Steric Blockade of Cas2/3 ssDNA Channel by β_2_-β_3_ Loop in AcrIF19. **a** Magnified view of the Cas2/3 region from structural alignment between Cas2/3-AcrIF19 and the *Vibrio* phage ICP1 Csy-dsDNA-Cas1-Cas2/3 complex (half form, PDB: 8K22). Rendered in cartoon representation; **b** Surface electrostatic potential representation of PatCas2/3, showing the relative positions of AcrIF19 and non-target ssDNA from the R-loop structure on the PatCas2/3 surface, as labeled directly in the panel. The ssDNA entry site is magnified in the inset; **c** Amino acid analysis of the loop region between β_2_ and β_3_ folds in AcrIF19; **d** Recruitment assay of AcrIF19-mediated inhibition of Cas2/3. Cas2/3 can be recruited by Csy-dsDNA, while the recruitment of the Cas2/3-AcrIF19 complex is significantly inhibited; **e** Analysis of interactions between the β_2_-β_3_ loop of AcrIF19 and the ssDNA entry channel of Cas2/3. Yellow dashed lines represent hydrogen bonds; red dashed lines indicate ionic bonds; **f** Recruitment assay of Cas2/3 with key amino acid mutations. Effects of wild-type (WT) AcrIF19 and mutations of residues (Y18, E19, D22, E23, and E27) within the β_2_-β_3_ loop involved in binding on the recruitment of Cas2/3. All samples were separated by electrophoresis using 5% TBE native gels (Native-PAGE). For (**d**) and (**f**), the experiments were repeated independently three times, with similar results.

The characteristic structural element of AcrIF19 within the AcrIF19-Cas2/3 complex, namely the β_2_-β_3_ loop, was comprehensively investigated structurally and functionally. First, we aligned the Cas2/3-AcrIF19 cryo-EM structure with the Vibrio phage ICP1 Csy-dsDNA-Cas1-Cas2/3 complex (half form, PDB: 8K22) for detailed analysis (Supplementary Fig. 3a). For this structure, our visualization focused on Cas2/3 and the R-loop. The Cas2/3-AcrIF19 structure aligns well with the complex’s Cas2/3, and AcrIF19’s binding site overlaps with that of non-target ssDNA (Fig. 3a). Surface charge analysis of ICP1 Cas2/3 revealed two distinct positively charged ssDNA-binding regions: one is the non-target ssDNA channel entrance, and the other is the HD domain’s nuclease catalytic site. First, analysis of the ssDNA channel entrance showed that the R-loop’s non-target ssDNA strand enters Cas2/3’s positively charged internal channel via an entrance, extending through this channel to the second positively charged region at the HD domain’s active site (Supplementary Fig. 3b). Notably, superimposing AcrIF19 onto the Cas2/3 structure revealed that it tightly embeds in the ssDNA entrance. Magnification of this entrance showed that AcrIF19’s β_2_-β_3_ loop inserts like a key into this ssDNA channel, completely occluding it (Fig. 3b). To investigate the basis for the specific insertion of the AcrIF19 loop into the ssDNA channel of Cas2/3, we analyzed the amino acid sequence of this loop and found it was composed primarily of polar amino acids—including glutamic acid (Glu), aspartic acid (Asp), tyrosine (Tyr), and serine (Ser) (Fig. 3c). Thus, we propose that the AcrIF19 loop competitively binding to the Cas2/3 ssDNA channel entrance to exclude endogenous ssDNA. This blocks Cas2/3 from recognizing non-target ssDNA in the Csy-dsDNA R-loop, thereby inhibiting Cas2/3 recruitment. EMSA demonstrated that while monomeric Cas2/3 is recruited by the Csy-dsDNA complex, the recruitment process is significantly impaired not only by pre-formed Cas2/3-AcrIF19 complexes but also by supplemental addition of AcrIF19 during the reaction (Fig. 3d and Supplementary Fig. 3c).

### AcrIF19 uses an integrated steric-electrostatic mechanism for Cas2/3 ssDNA channel occlusion

After confirming that the AcrIF19 β_2_-β_3_ loop spatially occupies the ssDNA-binding channel, we sought to elucidate the molecular basis of its inhibitory function. Interface analysis revealed extensive AcrIF19-Cas2/3 interactions: hydrogen bonds form between AcrIF19 Tyr18/Ser20 and Cas2/3 Glu1083, AcrIF19 Tyr21 and Cas2/3 Arg891/Lys896; additionally, salt bridges exist between AcrIF19 Glu19 and Cas2/3 Lys896, and between AcrIF19 Glu27 and both Cas2/3 Arg891 and Lys896. Additionally, the carboxylate oxygens of Glu27 (AcrIF19) form hydrogen bonds with the side-chain amine group of Lys902 (Cas2/3). Notably, Glu23 (AcrIF19) is positioned deepest within the Cas2/3 ssDNA channel, engaging in critical interactions: its backbone carbonyl oxygen forms a hydrogen bond with the hydroxyl group of Ser998(Cas2/3), while its side-chain carboxylate oxygens establish salt bridges with the guanidinium groups of Arg996(Cas2/3) (Fig. 3e). Subsequently, we identified two additional AcrIF19 homologs through BLAST analysis (both derived from *Pectobacterium parmentieri*; GenBank: WP_121304167.1 and WP_121268704.1), expanding our dataset to three homologs. Crucially, the Leu17-Tyr28 segment within the β_2_-β_3_ loop exhibited high conservation across all homologs, collectively suggesting a pivotal functional role for this residue (Supplementary Fig. 3d).

Based on the prevalence of acidic residues within the β_2_-β_3_ loop and their spatial positioning in the ssDNA channel, we engineered the AcrIF19 mutants (Y18F/E19R/D22N/E23R/E27R) to probe electrostatic complementarity. This construct replaces key acidic residues (Glu19, Glu23, Glu27) with arginine (E19R, E23R, E27R) to disrupt charge complementarity with Cas2/3, while Asp22 was conservatively substituted with asparagine (D22N) and Tyr18 with sterically conservative phenylalanine to maintain local conformation. In vitro DNA cleavage inhibition and Cas2/3 recruitment assays showed that charge-reversing mutations (E19R, E23R, E27R) severely impaired inhibitory activity, while structurally conservative substitutions (Y18F, D22N) only mildly reduced function, demonstrating that electrostatic complementarity mediated by acidic residues is crucial for competitive channel occlusion and effective Cas2/3 inhibition by AcrIF19 (Fig. 3e, f and Supplementary Fig. 3e). Taken together, AcrIF19’s β_2_-β_3_ loop competitively occupies the Cas2/3 ssDNA entrance, blocking Cas2/3 recognition of the Csy-dsDNA complex’s R-loop and thereby inhibiting recruitment.

### AcrIF19 C-terminal tail motif serves an anchoring role

**Fig. 4:**
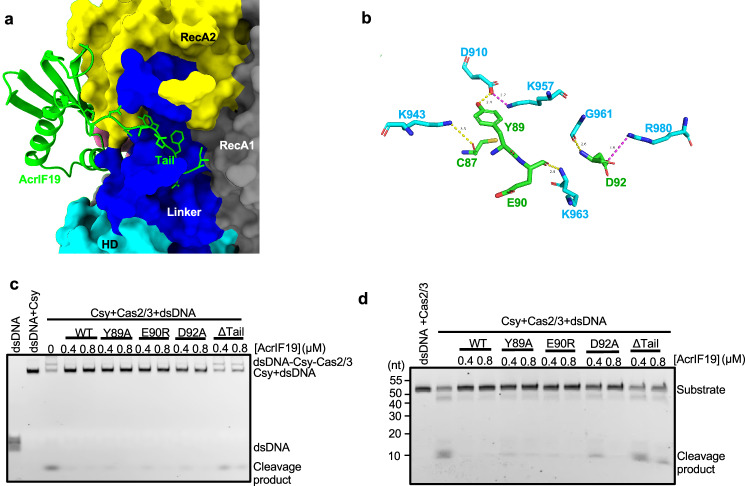
The C-terminal tail of AcrIF19 functions as an anchoring element. **a** Enlarged view of the Cas2/3-AcrIF19 complex focusing on the C-terminal tail of AcrIF19. AcrIF19 is shown in green cartoon representation, while Cas2/3 is depicted as a surface model with domains colored distinctly; **b** Interaction analysis between the tail structure of AcrIF19 and Cas2/3, yellow and red dashed lines represent hydrogen bonds and ionic bonds, respectively; **c**,**d** Effects of key amino acid mutations in the tail domain of AcrIF19 on Cas2/3 recruitment and dsDNA cleavage activity. For (**c**) and (**d**), the experiments were repeated independently three times, with similar results.

After elucidating the essential function of the β_2_-β_3_ loop in channel blockade, we further investigated whether the distinctive C-terminal tail of AcrIF19 contributes to its inhibitory function. Our magnified view of the AcrIF19 C-terminal tail adjacent to the Cas2/3 surface reveals that the Linker and RecA2 domains are the primary regions interacting with this tail (Fig. 4a). Specifically, the AcrIF19 tail engages Cas2/3 through key residues including Cys87, Tyr89, Glu90, and Asp92. These residues actively form hydrogen bonds with Cas2/3: Cys87 with Lys943, Tyr89 with Asp910, Glu90 with Lys963, and Asp92 with Gly961. Furthermore, a salt bridge is established between the carboxyl group of the Asp92 side chain and the amino group of the Arg980 side chain in Cas2/3 (Fig. 4b). To investigate the function of key residues in the AcrIF19 C-terminal tail, we generated single-point mutants and a tail-truncated mutant. After protein purification, these mutations were evaluated for their effects on Cas2/3 recruitment inhibition via EMSA. Results revealed that most single-residue substitutions did not significantly compromise AcrIF19-mediated inhibition. Strikingly, tail truncation resulted in nearly complete loss of inhibitory function (Fig. 4c). Consistently, using dsDNA cleavage assays, we found that these mutant proteins retained inhibitory activity. However, the D92A mutants exhibited partial loss of suppression, while the tail-truncated mutant showed nearly complete abolition of inhibitory function (Fig. 4d). MST affinity measurements revealed that the tail-truncated mutant (ΔTail) exhibited the largest increase in dissociation constant (*K*_d_) compared to other mutants, with a *K*_d_ value 101.7-fold higher than that of wild-type (WT) AcrIF19, indicating that the truncated region plays a critical role in mediating AcrIF19 binding to Cas2/3 (Supplementary Fig. 4d). Comparative analysis of the structures of *P. atrosepticum* Cas2/3-AcrIF19 (PatCas2/3-AcrIF19) and *P. aeruginosa* Cas2/3-AcrIF3 (PaeCas2/3-AcrIF3) revealed two mechanism-determining features: (1) The C-terminal tail of AcrIF19 docks into an electropositive pocket within the linker domain of PatCas2/3–a region exhibiting attenuated positive charge in PaeCas2/3; (2) Conformational clashes occur between the β_2_-β_3_ loop of AcrIF19 and the ssDNA entry channel of PaeCas2/3. These structural disparities collectively account for AcrIF19’s selective inhibition of PatCas2/3 over PaeCas2/3 (Fig. 4a, b, and Supplementary Fig. 4a–c). Collectively, our data establish the AcrIF19 tail as a molecular anchor essential for productive Cas2/3 engagement.

### AcrIF19 competitive binding occludes the catalytic channel and inhibits Cas2/3 nuclease activity

**Fig. 5:**
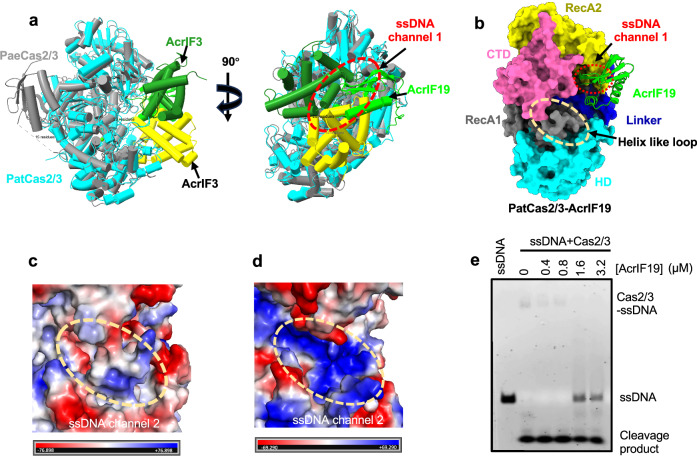
AcrIF19 inhibits the ssDNA nuclease cleavage activity of Cas2/3. **a** Structural alignment of the PatCas2/3-AcrIF19 complex with the PaeCas2/3-AcrIF3 complex. All components are colored according to the key within the panel; **b** Surface representation of the PatCas2/3-AcrIF19 complex structure. AcrIF19 is depicted in cartoon form, and the positions of ssDNA channel 1 and the helix-like loop are marked as indicated in the key within the panel; **c**,**d** Surface electrostatic potential representation of the PatCas2/3-AcrIF19 and PaeCas2/3-AcrIF3 complex structures, with magnified views and comparative analysis of the ssDNA channel 2 at the HD domain cleavage site; **e** EMSA experiments demonstrate that AcrIF19 inhibits the binding and cleavage of single-stranded DNA by Cas2/3, the experiments were repeated independently three times, with similar results.

Having established that AcrIF19 blocks Cas2/3 recruitment by occupying the ssDNA entry channel (Channel 1), we next asked whether it also directly suppresses the nuclease activity of Cas2/3. To gain mechanistic insights, we compared our Cas2/3-AcrIF19 structure with the Cas2/3-AcrIF3 complex. While AcrIF19 and the AcrIF3 dimer occupy partially overlapping sites at Channel 1, their binding modes are distinct (Fig. 5a). Most strikingly, comparative analysis revealed a critical difference at the catalytic site: in the Cas2/3-AcrIF19 structure, a helix-like loop within the RecA1 domain sterically blocks the cationic DNA-binding channel (Channel 2) adjacent to the HD-nuclease catalytic site. This density is absent in the Cas2/3-AcrIF3 structure, suggesting it is disordered or dynamic in that complex (Fig. 5b–d). This structural disparity implies that AcrIF19 binding is associated with a dramatic remodeling of the catalytic pocket.

To elucidate the functional significance of this occluded channel, we predicted the PatCas2/3-ssDNA (17 bp) complex structure using AlphaFold3, obtaining high-confidence models (ipTM = 0.73, pTM = 0.92). These predictions confirmed a continuous positively charged channel extending from the entry point to the catalytic core (Supplementary Fig. 5a–c). To validate the accuracy of our predicted structure, we further predicted a complex between PatCas2/3 and an 11-base ssDNA using AlphaFold3, which generated high-confidence models (ipTM = 0.84, pTM = 0.91), confirming the structural reliability of this complex (Supplementary Fig. 5d, e). Structural alignment of this predicted complex with the experimentally resolved *T. fusca* Cas2/3-ssDNA structure (PDB: 4QQW) and the predicted PatCas2/3-ssDNA (17 bp) revealed strong overall similarity, particularly at the ssDNA binding site, which strongly supports the reliability of our modeled complex (Supplementary Fig. 5f, g). Our structural observations revealed a dual blockade: AcrIF19 directly occupies Channel 1, and concurrently, its binding is associated with the steric occlusion of Channel 2. The ssDNA cleavage assays confirmed that AcrIF19 binding not only significantly inhibits the cleavage activity of Cas2/3 but also impairs its association with ssDNA (Fig. 5e). Meanwhile, MST assays separately determined the binding affinities of Cas2/3 to ssDNA and of the Cas2/3-AcrIF19 complex to ssDNA, revealing that AcrIF19 binding drastically compromises the binding of Cas2/3 to ssDNA, thus validating a competitive binding relationship between AcrIF19 and ssDNA (Supplementary Fig. 5h). Crucially, this inhibition of cleavage cannot be explained solely by the upstream blockage of Channel 1, as a theoretically open Channel 2 could still permit access to the catalytic site for pre-bound or alternatively-routed ssDNA. Therefore, the occlusion of Channel 2 must represent a direct and essential mechanism for abolishing nuclease activity. In conclusion, our data demonstrate that AcrIF19 binding achieves a dual-functional inhibition: it directly competes for ssDNA binding at the entry channel (Channel 1) to prevent recruitment, and it stabilizes the inhibitory conformation of the RecA1 loop, resulting in persistent occlusion of the catalytic channel (Channel 2). The molecular basis for this stabilization is investigated in the next section.

### AcrIF19 binding maintains Cas2/3’s autoinhibited state

**Fig. 6:**
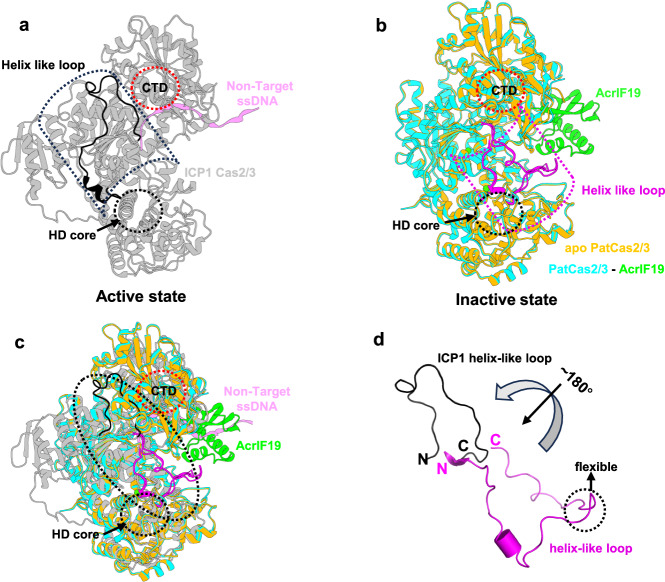
Conformational changes of a loop region (residues 488-523) in RecA1. **a** Relative positions of the helix-like loop in the *Vibrio* phage ICP1 Csy-dsDNA-Cas1-Cas2/3 complex (half-form, PDB: 8K22). ICP1 Cas2/3 and its helix-like loop are rendered in carton, with the relative positions of the CTD and the enzymatic active site of the HD domain are indicated by dashed lines; **b** Structural alignment of apo PatCas2/3 and the PatCas2/3-AcrIF19 complex. Apo PatCas2/3, PatCas2/3 in the PatCas2/3-AcrIF19 complex and AcrIF19 are shown in cartoon representation, with the helix-like loop highlighted and enclosed by a dashed box; **c** Structural alignment of apo PatCas2/3, the PatCas2/3-AcrIF19 complex and ICP Cas2/3 (recruited and activated by Csy-dsDNA) with the helix-like loops of all three structures are enclosed by black dashed lines; **d** Conformational changes of the loop region in the Cas2/3-AcrIF19 complex and the *Vibrio* phage ICP1 Csy-dsDNA-Cas1-Cas2/3 complex.

Structural analysis revealed that AcrIF19 binding is associated with steric occlusion of the HD domain’s catalytic channel (Channel 2) (Fig. 5b-d). In the AcrIF19-bound structure, the catalytic channel is stably blocked by a helical-like loop, maintaining Cas2/3 in an inactive conformation. To investigate the structural basis for this occlusion, we compared the Cas2/3-AcrIF19 complex with the activated Cas2/3 state captured in the Csy-dsDNA-Cas1-Cas2/3 complex (PDB: 8K22), wherein Cas2/3 is recruited by the R-loop of the Csy-dsDNA complex with ssDNA productively engaged in its channel. Global alignment revealed high overall similarity between the Cas2/3-AcrIF19 complex and the activated Cas2/3 state the latter complex (PDB: 8K22) (Supplementary Fig. 3a). However, a key conformational change was observed in RecA1: a helix-like loop (residues 488-523), which neighbors the CTD in the activated state (Fig. 6a), underwent stabilization upon AcrIF19 binding and inserted deeply into the catalytic channel, and interacted with the HD domain via Gln507 and the Gln511-Glu515 segment, as well as with the Linker domain via Gln493 and His496 (Fig. 6b, c and Supplementary Fig. 6a, b). Although residues 502-510 exhibited poorly resolved density, the well-defined trajectory of the loop unequivocally confirmed its inhibitory position (Supplementary Fig. 6c). This raised a critical question: Does AcrIF19 actively induce an inactive conformation, or does it stabilize Cas2/3’s intrinsic autoinhibitory state? To resolve this, we determined the structure of monomeric apo PatCas2/3 at 2.55 Å resolution (Supplementary Fig. 2a, b and Supplementary Fig. 6d). Its HD domain catalytic channel was similarly occluded by the helix-like loop, consistent with the AcrIF19-bound complex (Fig. 6b). This structural congruence demonstrates that AcrIF19 maintains Cas2/3’s preexisting autoinhibitory conformation without inducing structural rearrangements. Critically, Cas2/3 natively maintains an autoinhibited state that must undergo conformational transition to achieve catalytic activation during DNA cleavage. By comparing the structures of monomeric Cas2/3, the Cas2/3-AcrIF19 complex, and the activated Csy-dsDNA-Cas1-Cas2/3 complex (PDB: 8K22), we establish a key conformational transition in Cas2/3. This transition, triggered during R-loop formation/ssDNA recruitment, shifts the enzyme from an autoinhibited state—where the helix-like loop occludes the HD domain’s catalytic site—to an active state characterized by outward displacement of this loop, exposing the nuclease core. Structural prediction of Cas2/3 in complex with varying-length ssDNA further revealed that the helix-like loop adopts an open conformation displaced from the catalytic channel, illustrating Cas2/3’s dynamic activation process (Fig. 6d and Supplementary Fig. 5a, b, d). Taken together, our structural and functional analyses demonstrate that AcrIF19 maintains Cas2/3’s autoinhibited conformation by stabilizing the RecA1 helix-like loop within the catalytic channel, thereby preventing catalytic activity through steric occlusion while concurrently blocking substrate recruitment. This dual-functional suppression directly originates from AcrIF19 trapping Cas2/3 in its intrinsic autoinhibited state.

### AcrIF19 exerts dual-functional PatCas2/3 inhibition via a sequential mechanism

**Fig. 7:**
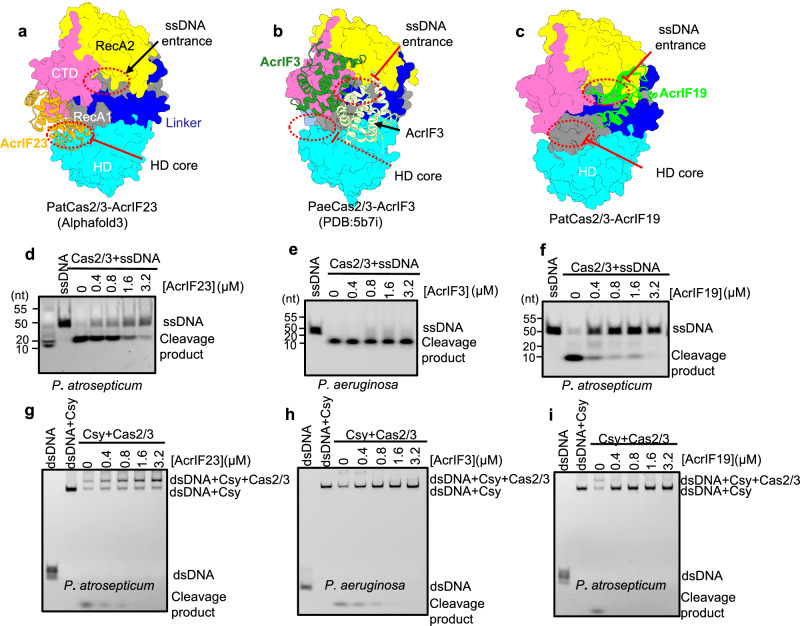
Comprehensive analysis of the structural features and inhibitory capabilities of AcrIF19, AcrIF23, and AcrIF3. **a–c** Relative binding positions of Anti-CRISPR proteins in the complex structures of PatCas2/3-AcrIF19, PatCas2/3-AcrIF23, and PaeCas2/3-AcrIF3; **d–f** ssDNA cleavage inhibition assays for AcrIF19, AcrIF3, and AcrIF23. AcrIF19 and AcrIF23 were tested with PatCas2/3, while AcrIF3 was assayed with PaeCas2/3, using 5ʹ-FAM-labeled Target ssDNA as the substrate in all reactions; **g–i** Cas2/3 recruitment inhibition assays for AcrIF19, AcrIF23, and AcrIF3. AcrIF19 and AcrIF23 were tested with PatCas2/3, while AcrIF3 was assayed with PaeCas2/3, using 5ʹ-FAM-labeled Target dsDNA as the substrate in all reactions. For (**d–i**), the experiments were repeated independently three times, with similar results.

To contextualize AcrIF19’s functional distinctiveness within anti-CRISPR mechanisms, we conducted comparative analyses against two established Cas2/3 inhibitors: AcrIF3 and AcrIF23. First, we evaluated the inhibitory mechanisms of these two specialized inhibitors. The herein reported structural analysis of AcrIF23 complex, for which no experimental structures are available, is based on a predicted structure obtained from AlphaFold3. AlphaFold3 prediction yielded a moderate-confidence model (ipTM = 0.65, pTM = 0.83), suggesting plausible binding at the catalytic channel (Channel 2) in Cas2/3’s HD domain (Fig. 7a, Supplementary Fig. 7a). While the ipTM score necessitates caution regarding the fine details of the interaction interface, mutational validation robustly confirms that conserved residues E25 and T26 form essential interfacial contacts (Supplementary Fig. 7b), wherein alanine substitution of these residues abrogates suppression activity^32^. Functional assays further demonstrated AcrIF23 exclusively inhibits ssDNA cleavage without impeding recruitment (Fig. 7d, g), validating the core prediction. In contrast, AcrIF3 functions as a dimer that sterically blocks the ssDNA entry channel (Channel 1). This blockade explains its potent inhibition of Cas2/3 recruitment and dsDNA cleavage, while exerting minimal effect on ssDNA cleavage (Fig. 7b, e, h). Thus, AcrIF3 and AcrIF23 employ complementary strategies: AcrIF3 mainly blocks initial Cas2/3 recruitment while AcrIF23 inhibits the catalytic cleavage step.

**Fig. 8:**
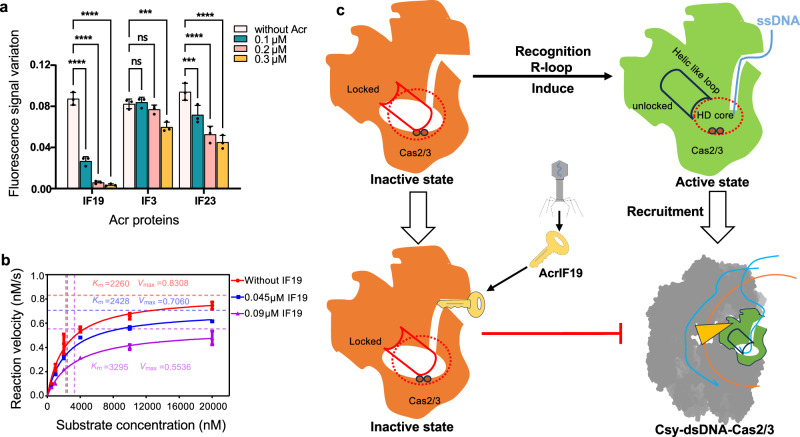
The Inhibitory Mechanism of AcrIF19. (**a**) Inhibitory Activity Comparison of AcrIF19, AcrIF3 and AcrIF23. The figure illustrates the inhibitory effects of three Acr proteins (AcrIF19, AcrIF3, AcrIF23) on the nuclease cleavage activity of Cas2/3 at 900 seconds across varying concentrations. Error bars indicate the mean ± SD (*n* = 3). *p < 0.05, ***p < 0.001, ****p < 0.0001, ns indicates no statistical significance. Significant differences were identified by One-way ANOVA followed by multiple comparison test. (**b**) Michaelis-Menten kinetic curves for Cas2/3 ssDNA cleavage activity in the absence (red) or presence of 0.045 μM (blue) or 0.09 μM (purple) AcrIF19. Reaction velocity (nM/s) is plotted against substrate concentration (nM). Fitted kinetic parameters (*K*_m_ and *V*_max_) are labeled for each condition. Error bars indicate the mean ± SD (*n* = 3). data are from three independent experiments (error bars represent SD); (**c**) Schematic model of AcrIF19 inhibitory mechanism. Cas2/3 is activated via R-loop binding and induce helix-like loop swing, enabling DNA cleavage and Csy complex recruitment. Phage-encoded AcrIF19 competitively binds Cas2/3 to block Csy recruitment, and its β_2_-β_3_ loop sterically occludes the ssDNA entry channel to suppress allosteric activation and DNA cleavage.

Next, we contrasted these with AcrIF19’s integrated mechanism. Structural analyses revealed that monomeric AcrIF19 uniquely disrupts both functional axes: its β_2_-β_3_ loop competitively occupies the recruitment channel (Channel 1), while binding stabilizes the autoinhibitory conformation of the RecA1 loop, physically blocking the catalytic channel (Channel 2) (Fig. 7c). This structural duality enables concurrent inhibition of Cas2/3 recruitment, ssDNA cleavage, and dsDNA cleavage (Fig. 7f, i), unifying the separate functions of AcrIF3 and AcrIF23. Systematic structural characterization further highlighted their evolutionary divergence: AcrIF19 (93 aa) contains a compact β-sheet core with a functional C-terminal tail; AcrIF3 (138 aa) is all-α-helical and requires dimerization; and AcrIF23 (156 aa) is predominantly α-helical (Supplementary Fig. 7c-e). Cross-species analysis revealed that while AcrIF23 suppresses ssDNA cleavage in both *P. atrosepticum* and *P. aeruginosa*, AcrIF3 demonstrates slight inhibitory activity in either system. In contrast, AcrIF19 selectively inhibits *P. atrosepticum* Cas2/3 without affecting the *P. aeruginosa* counterpart (Supplementary Figs. 7f and 8a–f). To compare the inhibitory effects of AcrIF3, AcrIF23, and AcrIF19 on the ssDNA cleavage activity of Cas2/3, we employed a FRET-based ssDNA cleavage assay using a FAM/BHQ dual-labeled reporter substrate^33^, with Cas2/3 nuclease activity quantified by fluorescence signal recovery. Results demonstrated that at equivalent concentrations, AcrIF19 exhibited the strongest inhibition of PatCas2/3 ssDNA cleavage, followed by AcrIF23 with intermediate efficacy, while AcrIF3 showed only marginal inhibitory activity even at higher concentrations (Fig. 8a). To further validate the dual inhibitory activity of AcrIF19, we utilized the same assay to determine the nuclease kinetic parameters of Cas2/3 in the absence and presence of AcrIF19, respectively. The results showed that AcrIF19 addition significantly increased the Michaelis constant (*K*_m_) but drastically reduced the maximum reaction rate (*V*_max_) of Cas2/3; these kinetic characteristics confirm that AcrIF19 acts as a mixed-type competitive inhibitor of Cas2/3 (Fig. 8b). In conclusion, our comparative profiling establishes that AcrIF19 is a unique anti-CRISPR protein capable of dual-functional inhibition of PatCas2/3. It achieves this not merely by combining two independent functions, but through a sequential mechanism wherein binding at the recruitment channel (Channel 1) competitively inhibits substrate recruitment and mediates catalytic site occlusion via stabilizing the autoinhibitory conformation of the RecA1 loop, defining a paradigm in CRISPR-Cas inhibition.

## Discussion

To our knowledge, most structurally characterized type I-F anti-CRISPR proteins inhibit immunity by directly engaging the Csy effector complex. The predominant mechanism involves blocking DNA binding, as employed by eleven characterized Acr proteins: six prevent target DNA hybridization with the crRNA guide (AcrIF1, AcrIF8, AcrIF9, AcrIF13, AcrIF14, AcrIF24), and five disrupt PAM recognition through direct or allosteric mechanisms (AcrIF2, AcrIF6, AcrIF7, AcrIF10, AcrIF11). AcrIF4 and AcrIF5 also bind Csy but uniquely disrupt Cas2/3 recruitment by targeting the Cas8f helical bundle essential for nuclease engagement, while AcrIF25 disassembles the Csy complex by dissociating Cas7f. Collectively, these well-characterized type I-F Acrs primarily function via direct Csy binding to disrupt specific immunity stages—a prevalence likely attributable to both the structural features of Csy and the complexity of the immune process. Given the indispensable role of Cas2/3 in DNA degradation, directly inhibiting its activity represents an efficient alternative strategy for Acrs.

Among the twenty-seven identified Acrs that inhibit the type I-F CRISPR-Cas system, the mechanism of AcrIF19 had remained unresolved. Here, we demonstrate that AcrIF19 specifically inhibits the nuclease activity of *P. atrosepticum* Cas2/3 in vitro by forming a stable complex—but not that of *P. aeruginosa* Cas2/3. Cryo-EM and biochemical analyses reveal a unique suppression mechanism: competitive occupation of the ssDNA entry channel (Channel 1) by the β_2_-β_3_ loop blocks R-loop recognition and prevents Cas2/3 recruitment to the Csy complex, which in turn stabilizes the autoinhibitory conformation of the RecA1 loop, physically obstructing the catalytic channel (Channel 2) to suppress nuclease activity (Fig. 8b, c). This sequential mechanism distinguishes AcrIF19 from all other type I-F Acrs. Notably, only two other Acrs are known to target Cas2/3 directly: AcrIF3 shares a partially overlapping binding site with AcrIF19, primarily at ssDNA channel entry 1 (Fig. 5a and Supplementary Fig. 9a), and sterically blocks this channel to prevent Cas2/3 recruitment and dsDNA cleavage. However, in contrast to AcrIF19’s robust suppression of both ssDNA and dsDNA cleavage, AcrIF3 exhibits significantly weaker inhibition of ssDNA cleavage. Structural analysis of the Cas2/3-AcrIF3 complex reveals that dimeric AcrIF3 binding leads to an open HD domain conformation (Fig. 5c). AcrIF23 binds to the HD domain, occupying the catalytic channel. Structural modeling reveals partial overlap between AcrIF23’s binding site and that of dimeric AcrIF3, with no such overlap observed for AcrIF19 (Supplementary Fig. 9b, c). This comparative analysis underscores that the sequential mechanism of Cas inhibition by AcrIF19 stands in contrast to the spatially distinct or partial mechanisms employed by AcrIF3 and AcrIF23. However, it is important to acknowledge limitations of this study. Key unresolved questions remain, including: (1) the precise mechanism by which AcrIF3 binding induces conformational changes in the Cas2/3 HD domain, particularly given the missing density for the helical-like loop; (2) the definitive structural details of the Cas2/3-AcrIF23 complex; and (3) whether additional Acrs exist that target the dynamic activation process of Cas2/3. These gaps highlight the need for follow-up studies focusing on high-resolution structural analysis of transient Cas2/3 states and systematic characterization of uncharacterized Acrs, which will deepen our understanding of Cas2/3 regulation and expand the landscape of type I-F Acr mechanisms.

In conclusion, our study elucidates the mechanism by which the key Cas2/3 nuclease is dynamically activated within the type I-F CRISPR-Cas system. Building on this discovery, we establish the molecular mechanism of the anti-CRISPR protein AcrIF19, which achieves inhibition by locking Cas2/3 in an inactive state. These findings collectively highlight the remarkable mechanistic diversity of anti-CRISPR proteins, redefine paradigms for Cas2/3 suppression, and unveil a mode of CRISPR-Cas inhibition.

## Methods

### Protein expression and purification

The *AcrIF19* gene was synthesized by GenScript and cloned into the pGEX-6p-1 vector to generate a GST-tagged fusion protein featuring a PreScission protease cleavage site between GST and AcrIF19; mutants were constructed via two-step polymerase chain reaction (PCR), subcloned, and purified following the wild-type protocol. Recombinant AcrIF19 protein was expressed in *Escherichia coli* BL21(DE3) cells induced with 0.2 mM IPTG at OD_600_ = 0.8, followed by 12–14 h of growth at 16 °C. Cells were harvested, resuspended in lysis buffer (1× PBS, 2 mM DTT, 1 mM PMSF), and lysed by sonication, with cellular debris removed via centrifugation at 18,000×g for 45 min at 4 °C. The clarified supernatant was applied to a self-packed glutathione Sepharose 4B column (GE Healthcare; 2 ml bed volume), washed with lysis buffer supplemented with 200 mM NaCl to remove contaminants, and subjected to on-column digestion using PreScission protease overnight at 4 °C to release AcrIF19 containing an N-terminal GST tag. The eluted protein was concentrated and further purified by size-exclusion chromatography (SEC) on Superdex 75 Increase column (GE Healthcare) pre-equilibrated with buffer containing 10 mM Tris-HCl (pH 8.0), 200 mM NaCl, and 5 mM DTT.

For Cas2/3 from *P. atrosepticum*, we extracted the genome of *P. atrosepticum* and designed primers based on the genomic information^34^. The full-length *Cas2/3* gene of *P. atrosepticum* was PCR-amplified, cloned into pET-28a (+) to generate N-terminal His_6_-tagged PatCas2/3, and expressed in *E. coli* BL21(DE3). Cultures (37 °C, with shaking) were induced with 0.2 mM IPTG at OD_600_ 0.6-0.8 (18 °C, 12–16 h). Cells were lysed by homogenization in 50 mM Tris, 300 mM NaCl, 10 mM imidazole, 1 mM PMSF (pH 8.0) buffer. Cleared lysate was purified by Ni-NTA chromatography (wash: 30 mM imidazole; elution: 300 mM imidazole) followed by SEC on Superdex 200 Increase column (10 mM Tris, 200 mM NaCl, 5 mM DTT, pH 8.0).

The Csy complex was purified as follows: *Cas8f/Cas5f and Cas7f/Cas6f*genes (*P. atrosepticum* genomic DNA) were PCR-amplified and cloned into pETDuet-1 and pACYCDuet-1, respectively; the crRNA cassette was inserted into pRSFDuet-1 plasmid and co-transformed into E. coli BL21(DE3). The Csy complex was purified sequentially by: Ni-affinity chromatography, anion-exchange chromatography (AEC), and SEC.

For the Cas2/3-AcrIF19 complex used for cryo-EM analysis, the *AcrIF19* gene was synthesized via PCR and cloned into the pET-22b (+) to generate a C-terminally untagged protein. This was co-transformed with Cas2/3-pET28a plasmid into *E. coli* BL21(DE3). Following co-expression of both plasmids, the complex was purified sequentially by Ni-affinity chromatography and SEC on Superdex 200 Increase column (GE Healthcare). The purified complex was flash-frozen in liquid nitrogen and stored at −80 °C. All purified proteins were validated by SDS-PAGE.

### Cryo-EM sample preparation and data acquisition

Aliquots of purified protein (4 μL at 2 mg mL⁻¹) were applied to glow-discharged Quantifoil R1.2/1.3 300-mesh copper grids (Quantifoil Micro Tools GmbH, Germany). Grids were blotted for 5.5 s and vitrified in liquid ethane using an FEI Vitrobot Mark IV operated at 8 °C and 100% relative humidity.

Cryo-EM data of Cas2/3-AcrIF19 complex were acquired on a 300 kV Titan Krios G3 microscope equipped with a Gatan K3 direct electron detector and a GIF Quantum energy filter (slit width -20 eV). Images were recorded automatically in super-resolution counting mode using E Pluribus Unum (EPU) software at a binned pixel size of 0.670 Å, with a total exposure of ~50 e^-^ Å^-^² fractionated over 32 frames. The nominal defocus range was -1.4 to -2.0 µm. Frame alignment and dose-weighted summation were performed with MotionCor2 (ref. ^35^).

Cryo-EM data of Cas2/3 in apo form were collected on a 300 kV Titan Krios G4 equipped with a Gatan K3 detector and a GIF Quantum energy filter (slit width of 20 eV). The defocus values ranged from -0.8 µm to -1.8 µm. Images were recorded automatically in super-resolution counting mode using E Pluribus Unum (EPU) software at a binned pixel size of 0.844 Å, with a total dose of about 50 e^-^ Å^-2^ over 32 frames. Frame alignment and dose-weighted summation were performed using the patch motion correction in CryoSPARC^36^.

### Cryo-EM data processing

The cryo-EM data processing workflow is summarized in Supplementary Fig. 1. All datasets were processed in CryoSPARC, with patch-CTF used to estimate contrast transfer function (CTF) parameters. For the Cas2/3-AcrIF19 complex, 2,320,821 particles were extracted from 4,183 micrographs. After iterative 2D classification, 566,834 particles were retained for further processing. Multi-class ab initio reconstruction and 3D classification identified a subset of 166,242 high-quality particles, which were subjected to non-uniform and local refinement to yield a 2.97 Å reconstruction. Using a similar data processing pipeline, 115,474 particles of Cas2/3 in the apo state were selected from 1996 micrographs, resulting in a 3D reconstruction at an overall resolution of 2.55 Å. Each final map was refined to C1 symmetry, and resolution was determined by gold-standard Fourier shell correlation at the 0.143 criterion. Data collection, refinement, and validation statistics are provided in Supplementary Table 1.

### Cryo-EM model building and refinement

Initial models were generated using AlphaFold3(ref. ^37^) and fitted into the cryo-EM densities with UCSF ChimeraX^38^. Model building was refined manually in Coot^37^, and the final model was optimized in real space using PHENIX^39^ with secondary structure and geometry restraints. Model quality was assessed by PHENIX, based on clashscore, MolProbity score, and Ramachandran statistics (Supplementary Table 1). All structural figures were prepared with PyMOL^40^ and UCSF ChimeraX.

### Double-stranded DNA preparation

Double-stranded DNA (dsDNA) substrates for electrophoretic mobility shift assays (EMSA) and in vitro DNA cleavage assays were prepared by annealing 5′-FAM (6-carboxyfluorescein)-labeled ssDNA oligonucleotides (Sangon Biotech) with unlabeled complementary strands at specified molar ratios: for EMSA, FAM-labeled *target* ssDNA was hybridized to unlabeled non-target ssDNA (1:1 ratio); for in vitro cleavage assays, FAM-labeled non-target ssDNA was hybridized to unlabeled target ssDNA (1:1.1 ratio).

*pae*Target DNA strand (54 bp; 5'-FAM fluorescein labeled or unlabeled)

GGAAGCCATCCAGGTAGACGCGGACATCAAGCCCGCCGTGAAGGTGCAGCTGCT

*pae*Non-Target DNA strand (54 bp; 5'-FAM fluorescein labeled or unlabeled)

AGCAGCTGCACCTTCACGGCGGGCTTGATGTCCGCGTCTACCTGGATGGCTTCC

*pat*Target DNA strand (54 bp; 5'-FAM fluorescein labeled or unlabeled)

GCAGTGAACTCTTTGTAATCACCTGTACGGCTGGCCCACACGGTTTCTAAGCTG

*pat*Non-Target DNA strand (54 bp; 5'-FAM fluorescein labeled or unlabeled)

CAGCTTAGAAACCGTGTGGGCCAGCCGTACAGGTGATTACAAAGAGTTCACTGC

### In vitro cleavage assay

dsDNA substrates were prepared by annealing target and non-target ssDNA strands (1.1:1 molar ratio) with 5'-FAM labeling on the non-target strand (BGI Synthesis). To assess AcrIF19/mutant activities, 0.32 μM Csy complex was pre-incubated with 0.1 μM dsDNA in cleavage buffer (20 mM HEPES pH 7.5, 100 mM KCl, 5% glycerol, 1 mM TCEP) at 37 °C for 10 min, while AcrIF19 variants (0.4, 0.8, 1.6, or 3.2 μM) were separately pre-incubated with 0.64 μM Cas2/3 under identical conditions. The mixtures were combined, supplemented with 0.8 μL metal ion cocktail (5 mM MgCl_2_, 75 μM NiSO_4_, 5 mM CaCl_2_, 1 mM ATP), and incubated at 37 °C for 20 min before quenching with 1% SDS/50 mM EDTA. Cleavage products were resolved on 14% urea-polyacrylamide gels (7 M urea) and visualized by fluorescence imaging. The identical assay methodology was employed for other Acr proteins within a concentration range of 0.4-3.2 μM.

For single-stranded DNA digestion, Acr proteins (0.4, 0.8, 1.6, or 3.2 μM final concentration) were pre-incubated with 0.64 μM Cas2/3 in reaction buffer (20 mM HEPES, pH 7.5, 100 mM KCl, 5% glycerol, 1 mM TCEP) at 37 °C for 30 min. Subsequently, 0.1 μM FAM-labeled ssDNA was added and incubated at 37 °C for 10 min. Reactions were terminated by adding 1% SDS/50 mM EDTA. The cleavage products were resolved on 14% urea-polyacrylamide gels (7 M urea) and visualized by fluorescence imaging.

### Electrophoretic mobility shift assay

For single-stranded DNA digestion, the reaction process was identical to the ssDNA cleavage experiment in vitro. After the reaction, the products were separated by electrophoresis using 5% TBE Native PAGE and visualized by fluorescence imaging.

To evaluate the effect of AcrIF19 and its mutants on Cas2/3 recruitment, we conducted Cas2/3 recruitment experiments. The experimental groups were designed to incubate with 0.1 μM dsDNA and 1.6 μM Csy complex in reaction buffer (20 mM HEPES, pH 7.5; 100 mM KCl; 1 mM TCEP; 5% glycerol) at 37 °C for 15 min. Subsequently, 0.4, 0.8, or 1.6 μM Cas2/3 were added to each reaction system, respectively, and incubated at 37 °C for 30 min. The total reaction volume for each group was 20 μL. The control groups, consisting of only dsDNA and the dsDNA/Csy complex, were used as references. After incubation, the samples were separated by 5% TBE native PAGE electrophoresis and visualized by fluorescence imaging.

For recruitment inhibition assays to assess the effects of AcrIF19 and its mutants on Cas2/3 recruitment, dsDNA substrates were prepared by annealing target and non-target ssDNA strands at a 1:1 molar ratio, with 5'-FAM labeling on the target strand (BGI Synthesis). First, 0.64 μM Cas2/3 and varying concentrations of AcrIF19 (final concentrations of 0.4 μM, 0.8 μM, 1.6 μM, or 3.2 μM) were added to reaction buffer (20 mM HEPES, pH 7.5; 100 mM KCl; 1 mM TCEP; and 5% glycerol), and incubated at 37 °C for 10 min. Subsequently, 0.8 μM Csy complex and 0.05 μM dsDNA were added to the mixture and incubated at 37 °C for 20 min. The reaction volume for each group was 20 μL. The control group, which was used as a reference, contained only dsDNA, dsDNA/Csy, and dsDNA/Csy/Cas2/3. Finally, the products were separated by 5% TBE Native PAGE and visualized by fluorescence imaging. Additionally, we employed an alternative approach for investigation. Unlike the above method, we first added 1.6 μM Csy complex and 0.1 μM dsDNA to the reaction buffer (20 mM HEPES, pH 7.5; 100 mM KCl; 1 mM TCEP; 5% glycerol) and incubated at 37 °C for 15 min. Subsequently, varying concentrations of Cas2/3 and Cas2/3-AcrIF19 (0.4, 0.8, or 1.6 μM) were added, and the mixture was incubated for 30 min. After the reaction, the products were separated by electrophoresis on 5% TBE Native PAGE and visualized by fluorescence imaging.

To assess the interaction between AcrIF19 and Csy-dsDNA complex, 0.8 μM Csy complex and different concentrations of AcrIF19 (0.4 μM, 0.8 μM, 1.6 μM, or 3.2 μM) were added to the reaction buffer (20 mM HEPES, pH 7.5; 100 mM KCl; 1 mM TCEP; 5% glycerol), and the mixture was incubated at 37 °C for 15 min. Subsequently, 0.1 μM dsDNA was added and incubated at 37 °C for 20 min. The reaction volume for each group was 20 μL. Groups containing dsDNA and dsDNA/Csy served as controls. After the reaction, the product was separated by electrophoresis using 5% TBE Native PAGE and the results were observed by fluorescence imaging.

### GST pull-down assay

To validate the interaction between AcrIF19 and Cas2/3, we performed a GST pull-down assay. Glutathione Sepharose beads (40 μL per tube) were washed three times with ultrapure water (500 μL) and equilibrated twice with equilibration buffer (50 mM Tris, 200 mM NaCl, 1 mM EDTA, 1% NP-40, 1 mM DTT, 10 mM MgCl_2_, pH 8.0). GST-AcrIF19 (15 μg) was incubated with beads for 10 min at room temperature to facilitate immobilization. Control tubes received an equal volume of protein-free buffer. After three washes with GST wash buffer (500 μL; centrifugation: 18,000×g, 1 min), the Cas2/3 (15 μg, untagged) was added to GST-AcrIF19-bound beads and incubated for 1 h at room temperature. The complex was subsequently washed four times with GST wash buffer. Beads were resuspended in 1× SDS loading buffer (15 μL) and analyzed by SDS-PAGE.

### FRET-based ssDNA nuclease activity assay

Enzyme kinetic experiments were conducted in EMSA buffer (containing 150 mM KCl, 20 mM HEPES, 5% (v/v) glycerol, 1 mM TCEP, pH 7.5): The final concentration of Cas2/3 nuclease was consistently set at 0.2 μM. Final concentrations of 0, 0.045, 0.09 μM AcrIF19. After incubation at 37 °C for 10 min, ssDNA substrates (ATATATAT, 5'-labeled with FAM, 3'-labeled with BHQ quencher) at final concentrations of 0.5, 1, 2, 4, 10, and 20 μM were added. Detection was performed using a real-time quantitative PCR instrument at 37 °C, acquiring fluorescence signals every 30 seconds for a total duration of 1200 seconds. Each concentration had 6 replicates, with a blank control included. The inhibitory effect of different AcrIF19 concentrations on Cas2/3 cleavage activity was quantified by calculating the fluorescence change relative to the blank control.

For comparison of the inhibitory activities of AcrIF19, AcrIF3, and AcrIF23, the concentration of Cas2/3 nuclease was maintained constant at 0.2 μM in the EMSA buffer, which contained 150 mM KCl, 20 mM HEPES, 5% v/v glycerol, 1 mM TCEP, and had a pH of 7.5. Acr proteins (AcrIF19, AcrIF3, AcrIF23) at final concentrations of 0.1 μM, 0.2 μM and 0.3 μM, respectively, were added to the above system, followed by incubation at 37 °C for 5 min. Finally, ssDNA molecules were supplemented to a final concentration of 2 μM. The reactions were performed on a quantitative real-time PCR instrument at 37 °C, with triplicate wells set for each concentration, and fluorescent readings were recorded every 30 seconds. For each group, the blank control value was subtracted from the fluorescence data obtained at the 900th second to calculate the fluorescence change, so as to compare the inhibitory effects of the three Acr proteins on the cleavage activity of Cas2/3 nuclease.

### Microscale thermophoresis assay

MST experiments were performed using a Monolith NT.115 instrument (NanoTemper). The target proteins PatCas2/3 and PatCas2/3-AcrIF19 were fluorescently labeled with RED-NHS fluorescent dye (Monolith NT™ Protein Labeling Kit RED) in a buffer system of 1×PBS containing 500 mM NaCl. The concentration of the fluorescently labeled proteins was fixed at 10 nM. Serially diluted ligands (Acr proteins and ssDNA) were subjected to 1:1 serial dilution starting from the highest concentration in the binding buffer (20 mM Hepes pH 7.5, 150 mM KCl, 5% v/v glycerol, 2 mM DTT), yielding 16 concentration points with a gradient ranging from 0.305 nM to 10 µM. The fluorescently labeled proteins at a fixed concentration were mixed with ligand solutions of varying concentrations in PCR tubes, and the mixtures were loaded into NanoTemper capillaries for detection. All measurements were performed at room temperature with the LED power set to 100% and MST power set to 40%. Each sample was measured in triplicate, and the mean values were adopted for subsequent analysis. Data analysis was conducted using NanoTemper Analysis Software.

### Reporting summary

Further information on research design is available in the Nature Portfolio Reporting Summary linked to this article.

## Supplementary information

Supplementary Information

Reporting Summary

Transparent Peer Review file

## Source data

Source data

## Data Availability

Cryo-EM density maps generated in this study have been deposited in the Electron Microscopy Data Bank (EMDB) under accession codes EMD-68883 (Cas2/3 in apo form) and EMD-66478 (Cas2/3-AcrIF19 complex). Atomic coordinates generated in this study have been deposited in the Protein Data Bank (PDB) under accession codes 23DJ (Cas2/3 in apo form) and 9X2F (Cas2/3-AcrIF19 complex). Structures of the Cas2/3-AcrIF3 complex (PDB: 5B7I), *Vibrio* phage ICP1 Csy-dsDNA-Cas1-Cas2/3 complex (half form, PDB: 8K22) and the *T. fusca* Cas2/3-ssDNA complex (4QQW) were referenced in the manuscript. All the unprocessed images and statistical data in this study can be found in the Source Data. Source data are provided with this paper.
