# Supplementary material for: Structural basis for dual mechanism of Cas2/3 nuclease inhibition by anti-CRISPR protein AcrIF19
Source: Nat Commun. 2026 May 19;17:6595. doi: 10.1038/s41467-026-73156-3 (PMC13381967; doi:10.1038/s41467-026-73156-3)
Supplement: Supplementary file 1 — Supplementary Information [file 41467_2026_73156_MOESM1_ESM.pdf]

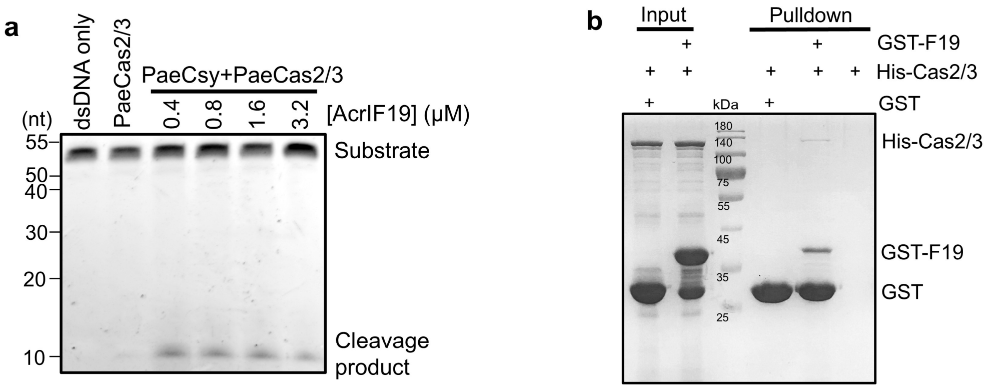

Supplementary Fig. 1 AcrIF19 does not inhibit the type I-F CRISPR-Cas system of *Pseudomonas aeruginosa.*

(a) Double-stranded DNA cleavage inhibition assay of AcrIF19; To investigate whether AcrIF19 can inhibit the double-stranded DNA cleavage activity of the I-F type CRISPR-Cas system in *Pseudomonas aeruginosa*. The concentrations of AcrIF19 are 0.4, 0.8, 1.6 and 3.2 μM; (b) Pull-down assay used to test the binding of AcrIF19 by the PatCas2/3. For a and b, the experiments were repeated independently three times, with similar results.

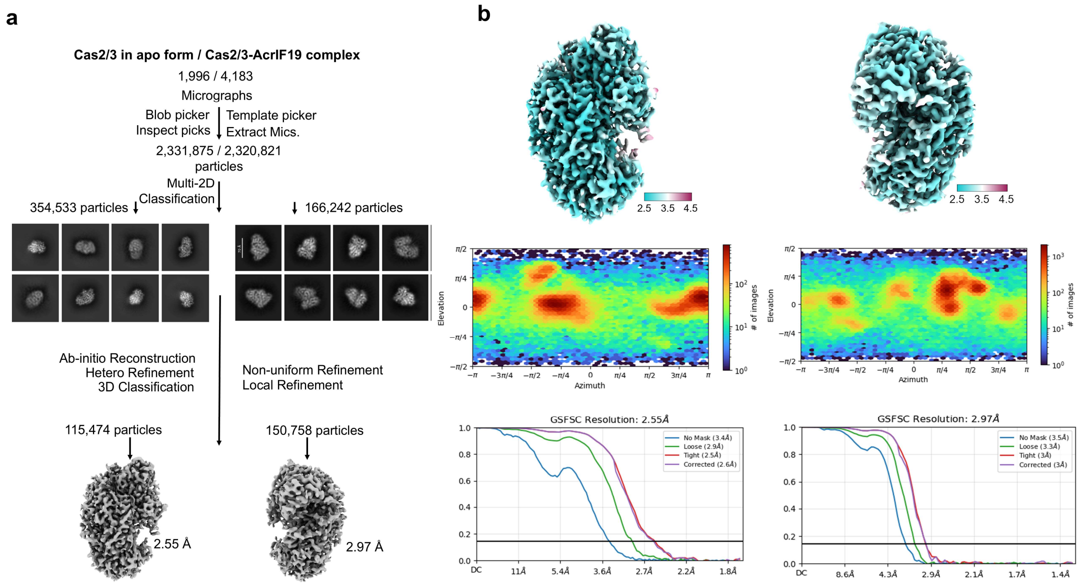

Supplementary Fig. 2 Data processing of cryo-EM dataset.

(a)Schematic outlining the data processing pipeline for the PatCas2/3-AcrIF19 complex and apo PatCas2/3;(b) Local resolution maps, angular distributions of particles contributing to the final reconstructions, and gold-standard Fourier shell correlation (FSC) curves from the 3D refinement of the PatCas2/3-AcrIF19 complex and Apo PatCas2/3, respectively.

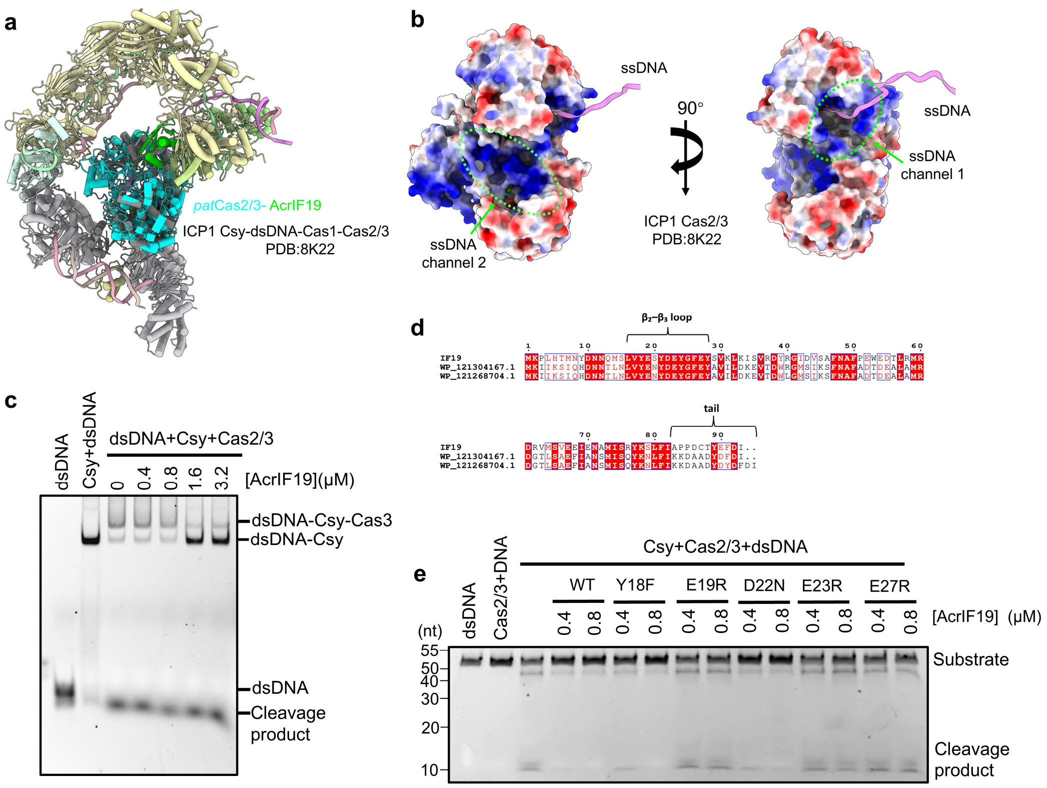

Supplementary Fig. 3 AcrIF19 inhibits the recruitment of Cas2/3.

(a) Structural alignment of PatCas2/3-AcrIF19 and ICP1 Csy-dsDNA-Cas1-Cas2/3. The cyan color represents the Cas2/3 of *P. atrosepticum*, and the green color represents AcrIF19; (b) Surface electrostatic potential map of Cas2/3 in the ICP1 Csy-dsDNA-Cas1-Cas2/3 complex. The pink band represents the non-target ssDNA within the R-loop structure, and the two dashed green circles indicate the positively charged regions of Cas2/3: ssDNA channel 1 and ssDNA channel 2; (c) Experiment on the inhibition of Cas2/3 recruitment by AcrIF19; (d) Sequence alignment analysis of AcrIF19 with two other homologous proteins (WP_121304167.1 and WP_121268704.1); (e) Assay of AcrIF19 (wild-type and mutants) inhibition of dsDNA cleavage. For c and e, the experiments were repeated independently three times, with similar results.

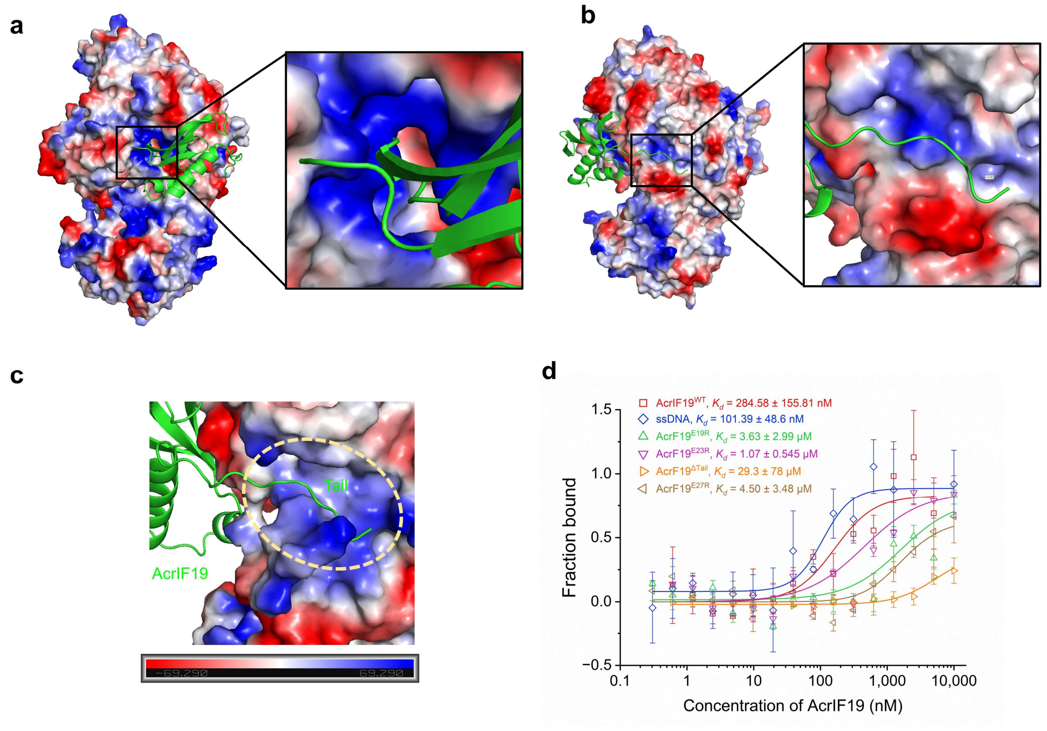

Supplementary Fig. 4 Structural basis for the lack of binding between AcrIF19 and PaeCas2/3.

(a) The loop between β2 and β3 in AcrIF19 exhibits a conformational conflict with the ssDNA channel entrance 1 of Cas2/3 derived from *P. aeruginosa*. (b) The C-terminal tail structure of AcrIF19 and the surface charge profile of the binding region with Cas2/3 derived from *P. aeruginosa*; (c) The C-terminal tail structure of AcrIF19 and the surface charge profile of the binding region with Cas2/3 derived from *P. atrosepticum*. The electrostatic potentials above were generated using PyMOL (Schrödinger, Inc.) with default parameters. Blue regions and red regions represent positive potential and negative potential, respectively; (d) Microscale thermophoresis (MST) assay measuring the binding affinities of ssDNA, AcrIF19 wild-type, and AcrIF19 mutants to Cas2/3. Fitted binding curves and calculated dissociation constants (K_d values) are indicated, with error bars representing the standard deviation (SD) from n = 3 independent measurements.

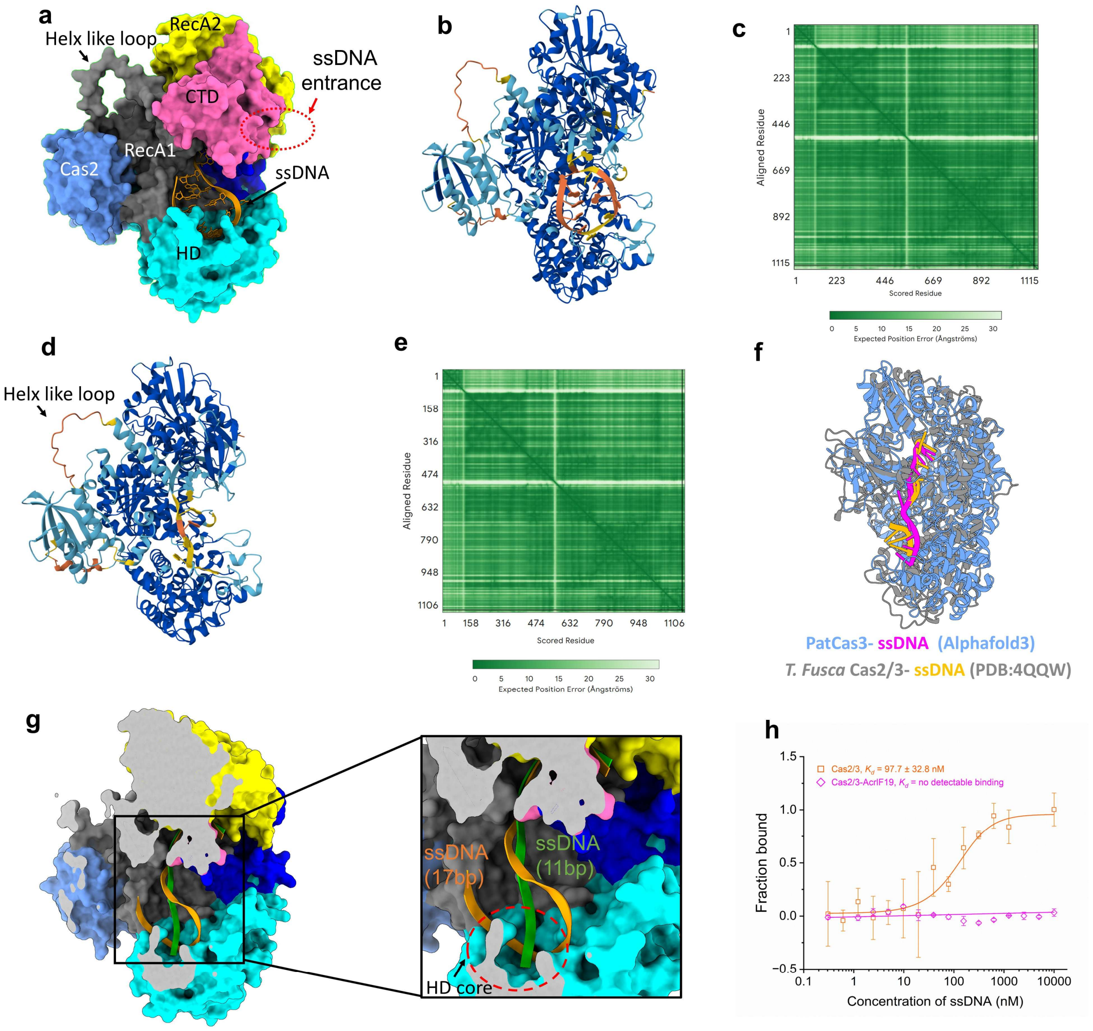

Supplementary Fig. 5 AcrIF19 inhibits the ssDNA nuclease cleavage activity of Cas2/3.

(a) Surface view of AlphaFold3-predicted PatCas2/3-ssDNA (17 bp); domains in distinct colors, with characteristic ssDNA entrance, helix-like loop and ssDNA pointed out by black arrows; (b, c) Structural prediction of the AlphaFold3-predicted PatCas2/3-ssDNA (17 bp) complex. Cartoon diagram, where color intensity indicates pLDDT-based prediction confidence(b), Corresponding Predicted Aligned Error (PAE) plots(c); (d, e) Structural prediction of the AlphaFold3-predicted PatCas2/3–ssDNA (11 bp) complex. Cartoon diagram, where color intensity indicates pLDDT-based prediction confidence(d), Corresponding Predicted Aligned Error (PAE) plots(e); (f) Structural alignment of predicted Cas2/3-ssDNA(11 bp) and *T. Fusca* Cas2/3- ssDNA (PDB:4QQW); (g) Structural alignment of predicted Cas2/3–ssDNA (11 bp/17 bp) complexes (surface representation); different domains in distinct colors, with cross-sectional views showing the two ssDNA binding positions; (h) MST assay of the binding affinity between ssDNA and Cas2/3/Cas2/3-AcrIF19. Fitted binding curves and calculated dissociation constants (K_d values) are indicated, with error bars representing the standard deviation (SD) from n = 3 independent measurements

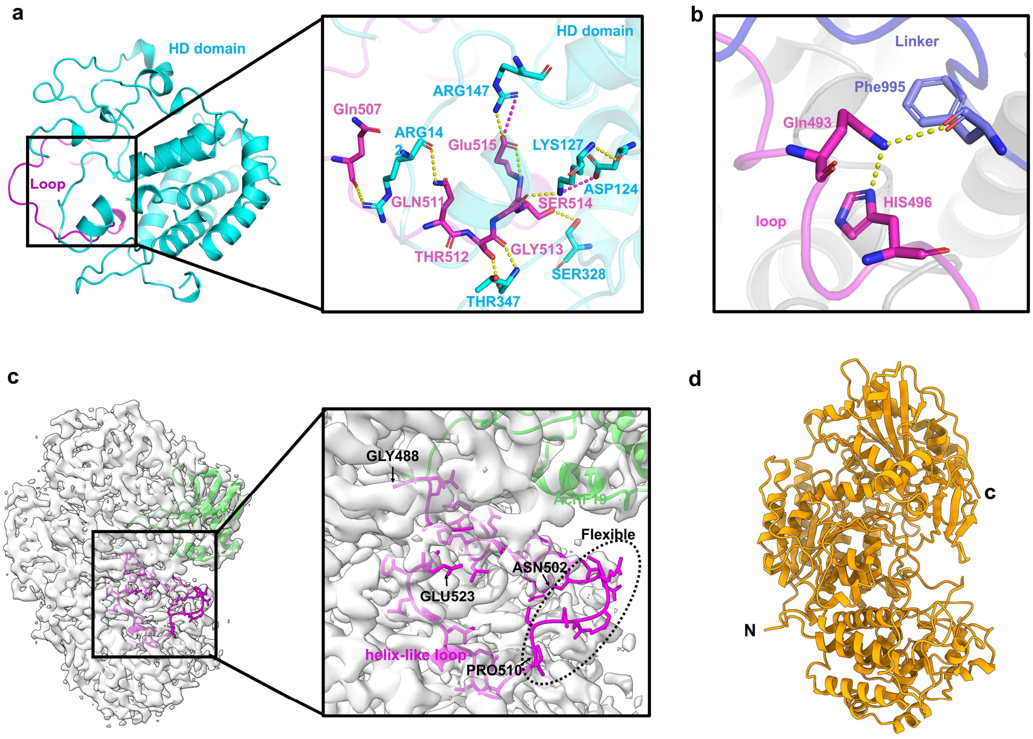

Supplementary Fig. 6 The position of the helix-like loop within the RecA1 domain after being locked in the Cas2/3-AcrIF19 structure.

(a) Analysis and enlarged view of the interaction between the helix-like loop and the HD domain. The HD domain is shown in blue, and the helix-like loop is shown in red. (b) Analysis of the interaction between the helix-like loop and the Linker domain. The hydrogen bonds and salt bridge interactions in the figure are shown as dashed lines; (c) Cryo-EM density map (contoured at 0.33σ, local resolution 2.7 Å) of the Cas2/3-AcrIF19 complex depicts the burgundy helical-like loop (residues 488-523) in RecA1 domain and green AcrIF19 inhibitor embedded within silver overall density; (d) Cartoon diagram of apo PatCas2/3 (orange), N- and C-termini labeled.

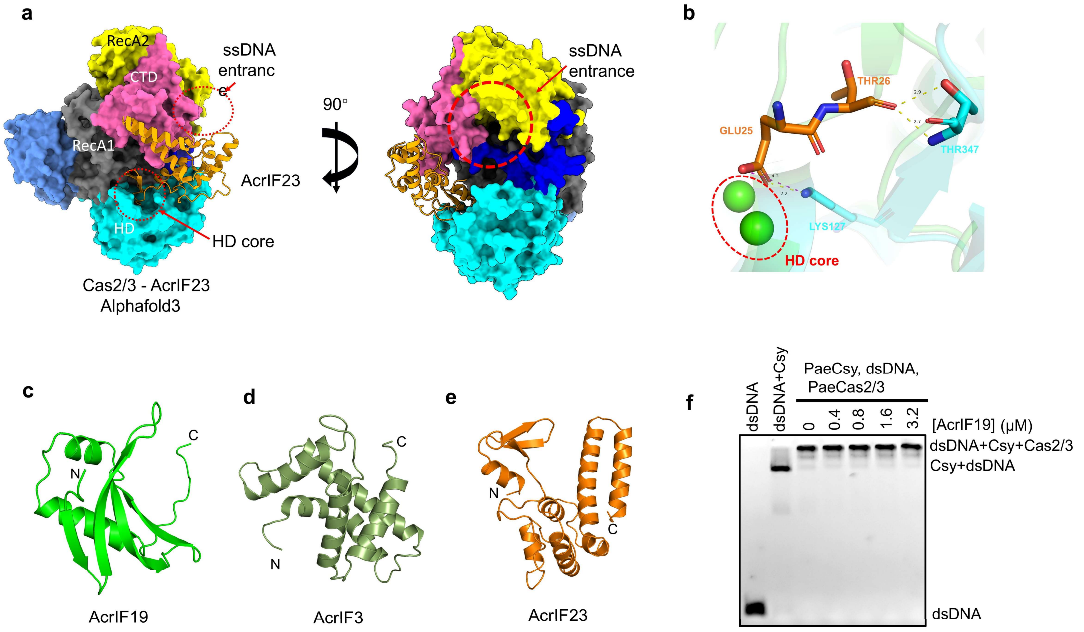

Supplementary Fig. 7 Structural comparison of AcrIF3, AcrIF23, and AcrIF19. (a) Alphafold3-predicted binding of AcrIF23 to PatCas2/3, with Cas2/3 shown as a surface representation (different domains colored distinctly) and AcrIF23 depicted as an orange cartoon. (b) Analysis of the interaction between AcrIF23 and the Cas2/3 HD domain based on the predicted structure. The hydrogen bonds and salt bridge interactions in the figure are shown as dashed lines; (c-e) The composition of secondary structural elements in AcrIF19, AcrIF3, and AcrIF23, respectively; (f) EMSA assay demonstrates that AcrIF19 does not inhibit the recruitment of *P. aeruginosa*-derived Cas2/3.

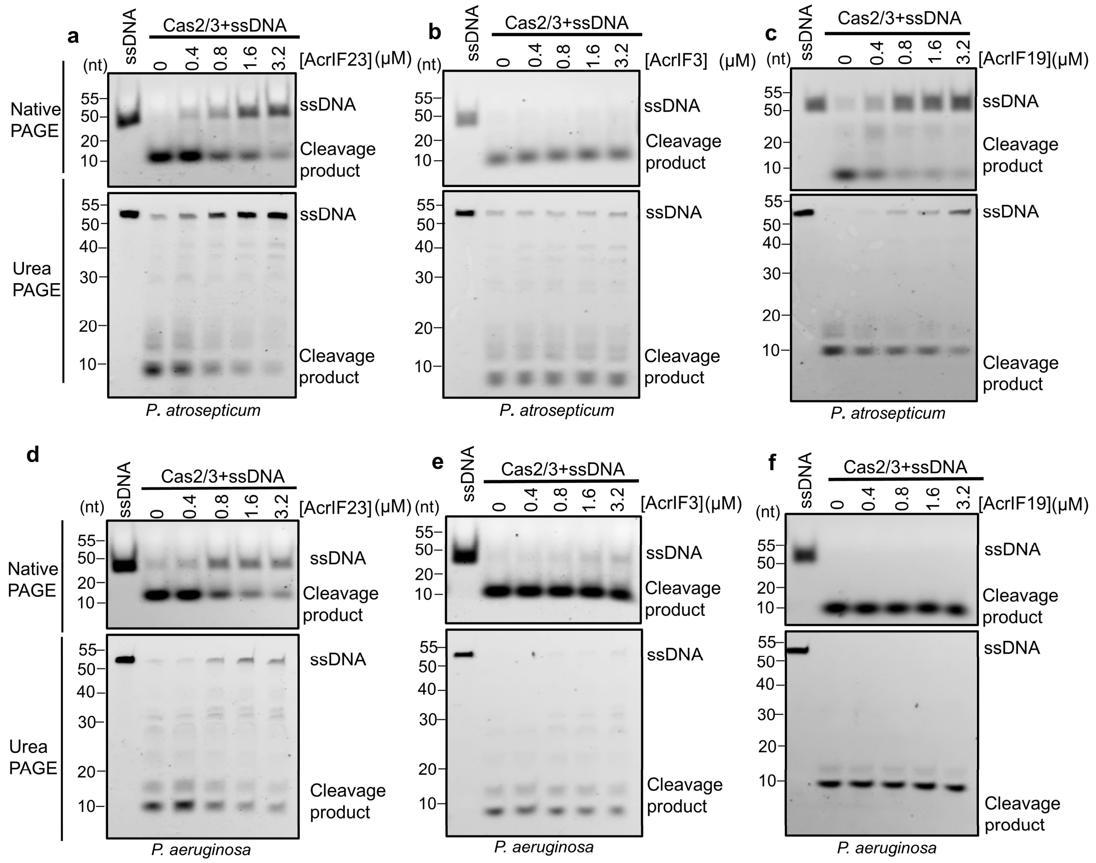

Supplementary Fig. 8 Comparison of the inhibitory effects of AcrIF23, AcrIF3, and AcrIF19 on the ssDNA cleavage activity of Cas2/3 from *P. atrosepticum* and *P. aeruginosa*.

(a-c) Inhibition of ssDNA cleavage by Cas2/3 derived from *P. atrosepticum* in the presence of AcrIF23 (a), AcrIF3 (b), and AcrIF19 (c), respectively. Comparisons were made using TBE native gels (top) and urea-denaturing gels (bottom); (d-f) Corresponding inhibition assays of ssDNA cleavage by Pseudomonas aeruginosa-derived Cas2/3 in the presence of AcrIF23 (d), AcrIF3 (e), and AcrIF19 (f), analyzed by TBE native gels (top) and urea-denaturing gels (bottom).

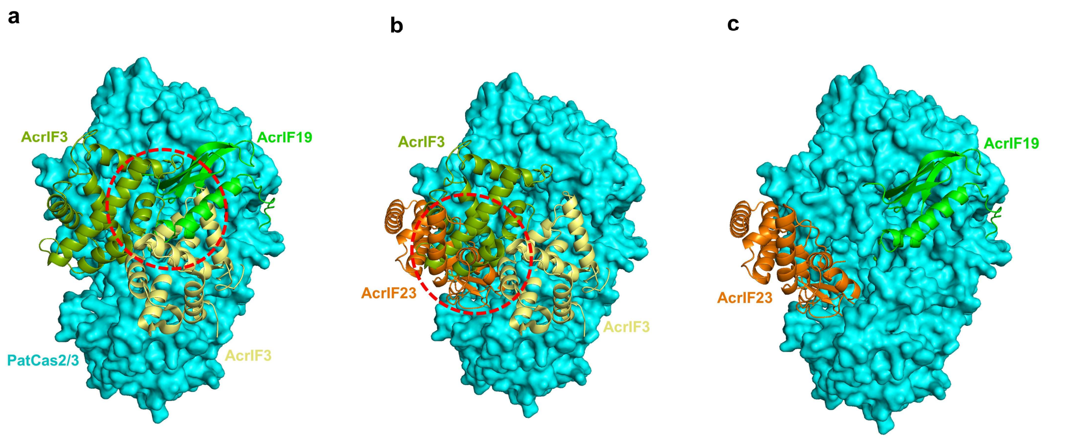

Supplementary Fig. 9 Binding interface comparison of AcrIF3/AcrIF19/AcrIF23 with PatCas2/3. Structural alignment of predicted PatCas2/3–AcrIF23, PatCas2/3–AcrIF19 and PaeCas2/3–AcrIF3 (PDB: 5B7I). PatCas2/3 (cyan, surface); Acr proteins (cartoon): AcrIF3, AcrIF23 and AcrIF19. (a) AcrIF3 vs AcrIF19; (b) AcrIF3 vs AcrIF23; (c) AcrIF23 vs AcrIF19 (relative positions).

Supplementary Table 1 | Cryo-EM data collection, refinement and validation statistics

	Cas2/3 in apo form (EMDB-68883) (PDB-23DJ)	Cas2/3-AcrIF19 complex (EMDB-66478) (PDB-9X2F)
**Data collection and processing**		
Magnification	105,000	130,000
Voltage (kV)	300	300
Electron exposure ($e^-/Å^2$)	50	50
Defocus range (μm)	-0.8 ~ -1.8	-1.4 ~ -2.0
Pixel size (Å)	0.844	0.670
Software	CryoSPARC-4	CryoSPARC-4
Symmetry imposed	C1	C1
Initial particle images (no.)	2,331,875	2,320,821
Final particles images (no.)	115,474	166,242
Map resolution (Å)	2.55	2.97
FSC threshold	0.143	0.143
Map resolution range (Å)	3.51-2.16	3.54 - 2.68
**Refinement**		
Initial model used	AlphaFold3	AlphaFold3
Model resolution (Å)	2.8	3.2
FSC threshold	0.5	0.5
Map sharpening *B* factor ($Å^2$)	67.9	134.0
Model composition		
Non-hydrogen atoms	7,914	8719
Protein residues	987	1083
Ligands	2	1
B factors ($Å^2$)		
Protein	50.55	57.90
Ligands	55.34	68.14
R.m.s deviations		
Bond length (Å)	0.002	0.003
Bond angles (°)	0.448	0.506
Validation		
MolProbity score	1.44	1.70
Clashscore	3.84	4.19
Poor rotamers (%)	1.18	1.71
Ramachandran plot		
Favored (%)	96.64	95.45
Allowed (%)	3.26	4.46
Disallowed (%)	0.10	0.09
