# Supplementary material for: Structural basis for dual mechanism of Cas2/3 nuclease inhibition by anti-CRISPR protein AcrIF19
Source: Nat Commun. 2026 May 19;17:6595. doi: 10.1038/s41467-026-73156-3 (PMC13381967; doi:10.1038/s41467-026-73156-3)
Supplement: Supplementary file 3 — Transparent Peer Review file [file 41467_2026_73156_MOESM3_ESM.pdf]

nature portfolio

Peer Review File

Structural basis for dual mechanism of Cas2/3 nuclease inhibition by anti-CRISPR protein AcrIF19

Corresponding Author: Professor Yongchao Xie

This file contains all reviewer reports in order by version, followed by all author rebuttals in order by version.

Version 0:

Reviewer comments:

Reviewer #1

(Remarks to the Author)
The manuscript "Structural Basis for Dual-Pathway Inhibition of Cas2/3 Nuclease by Anti-CRISPR Protein AcrIF19" by Sa et al. describes a structural and functional study of how the anti-CRISPR protein AcrIF19 inhibits Type I-F CRISPR immunity. Class I CRISPR systems are widespread, and 27 anti-CRISPR proteins targeting Type I-F CRISPR proteins have been identified. The mechanisms of most of these proteins are known; only a few remain functionally mysterious. This study reveals that, like only two other anti-CRISPR proteins (AcrIF3 and AcrIF23), AcrIF19 binds and inhibits the Cas2/3 nuclease protein rather than the the Csy complex. The authors show that AcrIF19 acts in two ways: first by occluding a binding site for ssDNA generated by the Csy complex, and second by allosterically inducing conformational changes that prevent nuclease activity.

Overall, this study is well done, and the writing is clear. In general, I support publication after the authors address some questions and concerns. Apart from specific feedback (below), one major point is that the figures are difficult to interpret. This is especially true for figures that compare how different anti-CRISPRs bind and inhibit Cas2/3. The figure describing the allosteric inhibition mechanism through movement of the "helix-like loop" motif is the most difficult to understand.

Specific points:

Introduction, lines 79-81: The sentence "Notably, only AcrIF3 and AcrIF23 suppress Cas2/3 recruitment or enzymatic activity through direct binding" is misleading, since it implies that these are known to be the only two proteins that act though this mechanism. I suggest rephrasing to "Notably, only AcrIF3 and AcrIF23 have been shown to suppress Cas2/3 recruitment or enzymatic activity through direct binding".

Results, line 157-168: I do not agree with the authors that the AcrIF19 beta-2 beta-3 "mimics ssDNA". I do not dispute that AcrIF19 occludes the ssDNA binding site on Cas2/3, and uses both negatively charged and aromatic residues to do so. But, the authors would need to demonstrate that these residues form structurally equivalent interactions with Cas2/3 as are observed in ssDNA complexes to support this strong claim. I suggest the authors rephrase their claims to simply state that AcrIF19 occludes the ssDNA binding site and thereby prevents ssDNA binding by Cas2/3.

Lines 175-176: The notation of amino acids interacting between AcrIF19 and Cas2/3 is confusing. Can the authors provide an indication here of which protein they are referring to in each case (as in the following sentences, where this is noted)?

Results section: how many examples of AcrIF19 homologs are available? It would be helpful to show that the residues the authors discuss and mutate (in the beta-2 beta-3 loop and the C-terminus) are highly conserved in this family of proteins.

Lines 281-296: The discussion of how the Cas2/3 "helix-like loop" moves in response to AcrIF19 binding, and the accompanying figure, is confusing. How do the authors know that this loop changes position upon AcrIF19 binding, since they are comparing the structure to an uninhibited structure of a completely different Cas2/3 protein? From Figure 6a, it looks like the position of the helix-like loop in the ICP1 Cas2/3 protein would be impossible for the PatCas2/3 helix-like loop, since part of the folded PatCas2/3 protein overlaps this loop's position in ICP1? Please explain and show this more clearly.

Lines 298-306: The comparison of inhibition mechanisms between AcrIF3 and AcrIF19 suffers from the same problem as

above. The authors do not have an unliganded structure of PatCas2/3, so they are comparing to a homologous protein. How do the authors know that the structural differences they highlight are due to AcrIF3/19 binding, not simply different between the two Cas2/3 proteins being compared?

Please add PAE diagrams and pTM/ipTM scores for all AlphaFold predictions.

Lines 322-324: Please explicitly note here that the "structural analysis" being discussed is based on a predicted structure, not an experimental structure. How confident is this structure?

Figures:

Figure 1a/b: the AcrIF19 concentrations used in these assays are not noted in either the figure itself or the figure legend. In the current figure, the text "(uM)" above each gel is not informative, because there are no numbers for AcrIF19 concentration (only a triangle indicating increasing concentrations). The same problem exists in Extended Data Figure 1a.

Figure 2a: It would be helpful for a reader if the authors included a domain diagram (from N to C terminus) of the different domains of Cas2/3, and the colors of each domain as used in this panel. I see the authors included exactly this panel as Extended Data Figure 2e - can this be moved to the main text figures?

Figure 2: It would be helpful if the authors labeled both the "loop" and "tail" elements in panels b and c, in addition to panel e.

Figure 5e: Please add labels on the figure, or list in the figure legend, the concentrations of each component used in this assay.

Reviewer #2

(Remarks to the Author)
This is an interesting, comprehensive structural biology study that reports the cryo-EM structure of the Pectobacterium atrosepticum Cas2/3–AcrIF19 complex at 2.97 Å resolution. The work identifies a dual inhibitory mechanism in which AcrIF19 simultaneously blocks Cas2/3 recruitment and nuclease activity through ssDNA mimicry and allosteric conformational locking of the RecA1 loop. The manuscript is methodologically rigorous, mechanistically insightful, and broadly relevant to CRISPR–Acr research.
However, the presentation can be significantly improved by clarifying novelty relative to AcrIF3 and AcrIF23, tightening structural interpretations, and providing clearer biochemical validation of the proposed allosteric effect. While the concept of a dual mechanism is intriguing, more rigorous and direct evidence is required to clearly substantiate this claim.

Major Comments:
1. The current text briefly notes that AcrIF3 blocks recruitment and AcrIF23 inhibits cleavage, but the discussion should compare binding interfaces and functional overlaps more directly.
2. The term "dual-pathway inhibition" is conceptually strong. It is needed to clarify whether this refers to simultaneous inhibition of (i) recruitment and (ii) catalysis, or to two structurally distinct blockade routes.
3. Figure 6A and 6B: The authors claim that the helix-like loop in the RecA1 domain undergoes a 180° rotation upon binding of AcrIF19, based on structural comparison with Cas2/3 homologs from other species. However, it remains unclear whether this structural difference is species-specific or indeed induced by AcrIF19 binding. To support this claim, it would be important to determine the structure of apo PatCas2/3 (i.e., in the absence of AcrIF19). Since the current structure was determined by cryo-EM, it seems feasible to obtain an apo-state reconstruction by simply excluding AcrIF19 during sample preparation. Such verification appears necessary to support the claim of dual inhibition.
4. The authors should provide quantitative ssDNA cleavage kinetics showing that AcrIF19 disrupts catalysis beyond steric blocking
5. Cryo-EM map quality for the remodeled RecA1 loop (residues 488–523) is described as "partially unresolved"; representative density maps should be included to verify model confidence.

Minor Comments
1. β_2–β_3 sometimes written as β2–β3,
2. 2mg/mL or 2mgmL-1
3. Reference21 is not related to Acr. Please check it again.

Reviewer #3

(Remarks to the Author)
This study investigates the inhibitory mechanism of the previously discovered anti-CRISPR protein AcrIF19, which inhibits the type I-F CRISPR-Cas system of Pectobacterium atrosepticum. This system is composed of a multi-subunit complex that binds targeted dsDNA and recruits the helicase-nuclease Cas2/3 for target degradation. The authors use in vitro DNA cleavage assays and Cas2/3 recruitment assays, and cryoEM analysis to uncover the structural basis for inhibition by IF19. They find that IF19 occupies two important sites within Cas2/3. First, IF19 fits snugly into the same channel that Cas2/3 uses to bind its non-target ssDNA substrate, and the authors identify a loop important for this role. Secondly, the C-terminal tail of IF19 occupies and inhibits the HD nuclease active site of Cas2/3.

Overall the findings are interesting and the experiments well-executed. However, the authors do overstate their conclusions at times, with more experimental data required to substantiate their claims.

1. The authors repeatedly frame AcrIF19 as having dual mechanisms of inhibition – it both blocks the ssDNA entry channel and blocks the HD active site responsible for ssDNA cleavage. They nicely show the counterexamples of AcrIF3 (which inhibits Cas2/3 recruitment but not ssDNA cleavage) and AcrIF23 (which does the opposite). However, though the authors comment on it in Lines 259-262, the separability of these dual mechanisms is not experimentally demonstrated for AcrIF19, in fact, some experimental conclusions seem to argue against it. If each mechanism were sufficient for Cas2/3 inhibition, then mutations abolishing each of the two mechanisms individually should not abolish inhibitory activity, as the other activity could compensate. Furthermore, the effects of these individual mutations might be expected to influence only the impacted arm of the pathway, i.e. Cas2/3 recruitment or ssDNA cleavage. Instead, the authors demonstrate that both (i) charge reversal mutations in the loop occupying the ssDNA entry channel, and (ii) deletion of the C-terminal tail that inhibits the HD active site, abolish both Cas2/3 recruitment and ssDNA cleavage. The total loss of activity by both sets of mutations argues for a unified inhibition mechanism rather than a dual one. It is perhaps most easily explained by a model in which both the loop and the tail are required for stable binding of AcrIF19 to Cas2/3, and interaction experiments along these lines could be informative. Until the authors demonstrate some separability of these two activities, their conclusion of a dual inhibitory mechanism is unsupported.

2. The authors conclude that the loop of AcrIF19 is a "structural mimic" of ssDNA. This case is much less supported than other established examples of structural mimicry, where a protein exhibits the same fold and charge distribution as another macromolecule, e.g. PAM-containing DNA mimicry by AcrIIA4. In the case of the AcrIF19 loop, the authors argue that because the loop contains negatively charged residues and occupies the ssDNA entry channel, it is a mimic of ssDNA. By this logic, any drug that occupies an enzyme's active site could also be considered a mimic of the enzyme's substrate. I recommend that the authors eliminate the term "mimic" and instead refer to AcrIF19 as a competitive inhibitor of ssDNA binding. Further data to support this conclusion could also come from testing whether positively charged residues contacted by AcrIF19 in the ssDNA entry channel are required for DNA binding and cleavage by Cas2/3.

3. In the abstract, and throughout the manuscript, the authors claim that AcrIF19 represents the first Acr with a "dual-pathway" inhibitory mechanism. This is not true, as it was previously shown that AcrIIA1 inhibits Cas9 via direct binding to the HNH domain and via induction of Cas9 degradation, and that these are separable activities (see Osuna et al. 2020 Cell Host & Microbe). This claim of priority is unnecessary and needs to be removed.

4. The introduction is longwinded (paragraph 1 especially), with superfluous discussion about all the different CRISPR classes and types, adaptation and crRNA processing, which have been covered extensively elsewhere and are not really the focus of this manuscript. With the space made by this trimming, the authors should include more information regarding Cas2/3's ssDNase activity, which does feature centrally in the manuscript. Please explain the role of the ssDNase activity and how this results in degradation of dsDNA (Line 68).

5. Fig. 1 and Extended Data Fig. 1: Legends say AcrIF19 was added with the indicated concentrations. The concentrations are not indicated, there is only a triangle. Also relevant for Fig. 3d, 5e,7d-i. In all cases, please indicate concentrations or molar ratios of all Cas proteins / Acrs / substrates.

6. Fig. 1c: Please show the tract of Cas2/3 alone (with no Acr) as this is the relevant control trace. The Csy+AcrIF19 trace shown is uninformative.

7. I do not think it is required for this manuscript, but in general for future DNA cleavage assays, a non-targeted control substrate would be helpful.

8. Fig. 2b-c: Please label the Loop and Tail of AcrIF19.

Version 1:

Reviewer comments:

Reviewer #1

(Remarks to the Author)
The authors have done a good job addressing all reviewer questions. I support publication.

Reviewer #2

(Remarks to the Author)
The authors have resolved the apo structure of Cas2/3 and proposed a new mechanism based on it, adequately addressing all of my previous comments. My concerns have been fully resolved, and I recommend the manuscript for acceptance.

Reviewer #3

(Remarks to the Author)
The revised manuscript is much improved, with additional experimental data and appropriately toned down conclusions. I support publication and congratulate the authors on an excellent study.

Reviewer #4

(Remarks to the Author)
The paper studies AcrIF19, an anti-CRISPR protein that inhibits the type I-F CRISPR-Cas system in Pectobacterium atrosepticum by targeting Cas2/3, the nuclease-helicase that carries out DNA degradation. The main claim is that AcrIF19 is unusual because it blocks both recruitment of Cas2/3 to the target DNA complex and its nuclease activity once bound. The authors support this with cryo-EM, mutagenesis, binding assays, cleavage assays, and comparisons with other anti-CRISPRs.

The main result of the paper is that AcrIF19 binds directly to Cas2/3 rather than the Csy surveillance complex. Structurally, AcrIF19 uses two sections of the protein:

1.- A beta2-beta3 loop that plugs the ssDNA entry channel on Cas2/3, blocking access of the non-target DNA strand and impairing Cas2/3 recruitment

2.- A C-terminal tail that acts as an anchor to stabilize binding to Cas2/3.
The paper argues that this binding also keeps a RecA1 “helix-like loop” in a position that blocks the catalytic channel near the HD nuclease site, preventing DNA cleavage. In the revised manuscript, the authors frame this as an integrated cascade dual-function inhibition mechanism rather than two independent pathways.

After analyzing the maps and models and the claims in the paper, I think the authors should revised D22 in the beta2-beta3 loop, as it seems the side chain is out of teh density. The authors can adjust he residue but I think this may change some of teh contacts that they have described in the corresponding figure. I think the main conclusion is still fine.

However, the authors have what I think is 1-2 cations bound between His217, His 216, His361, D124, D324. I think this is the active center of the HD domain. Previous structures have modeled the density as Mn, but could be Ni which was used in the purification. I think the authors should check, whether the isolated Cas2/3 nuclease activity its OK. In a case of bad luck it could be that the lack of cleavage that they observed is due to an inhibition of the nuclease by a cation that binds and does not favor catalysis.

For HD nucleases, the effect of Ni is enzyme-specific some HD-domain enzymes are active with that cation and some show reduced activity compared with Mn and Fe. In some cases Ni can act as a poor substitute or become inhibitory depending on concentration and competition with the native metal.

Version 2:

Reviewer comments:

Reviewer #4

(Remarks to the Author)
The explanation of the authors is fine. I think the paper is ready for publication

Open Access This Peer Review File is licensed under a Creative Commons Attribution 4.0 International License, which permits use, sharing, adaptation, distribution and reproduction in any medium or format, as long as you give appropriate credit to the original author(s) and the source, provide a link to the Creative Commons license, and indicate if changes were made.
In cases where reviewers are anonymous, credit should be given to 'Anonymous Referee' and the source.
The images or other third party material in this Peer Review File are included in the article's Creative Commons license, unless indicated otherwise in a credit line to the material. If material is not included in the article's Creative Commons license and your intended use is not permitted by statutory regulation or exceeds the permitted use, you will need to obtain permission directly from the copyright holder.
To view a copy of this license, visit https://creativecommons.org/licenses/by/4.0/

Reviewer comments

Reviewer #1 (Remarks to the Author):

The manuscript "Structural Basis for Dual-Pathway Inhibition of Cas2/3 Nuclease by Anti-CRISPR Protein AcrIF19" by Sa et al. describes a structural and functional study of how the anti-CRISPR protein AcrIF19 inhibits Type I-F CRISPR immunity. Class I CRISPR systems are widespread, and 27 anti-CRISPR proteins targeting Type I-F CRISPR proteins have been identified. The mechanisms of most of these proteins are known; only a few remain functionally mysterious. This study reveals that, like only two other anti-CRISPR proteins (AcrIF3 and AcrIF23), AcrIF19 binds and inhibits the Cas2/3 nuclease protein rather than the Csy complex.

The authors show that AcrIF19 acts in two ways: first by occluding a binding site for ssDNA generated by the Csy complex, and second by allosterically inducing conformational changes that prevent nuclease activity.

Overall, this study is well done, and the writing is clear. In general, I support publication after the authors address some questions and concerns. Apart from specific feedback (below), one major point is that the figures are difficult to interpret. This is especially true for figures that compare how different anti-CRISPRs bind and inhibit Cas2/3. The figure describing the allosteric inhibition mechanism through movement of the "helix-like loop" motif is the most difficult to understand.

We appreciate the reviewer's high evaluation of our work and constructive suggestions. We have addressed all of the comments listed below with additional experiments and have revised the manuscript accordingly, which we believe have significantly improved our manuscript.

Specific points:

1. Introduction, lines 79-81: The sentence "Notably, only AcrIF3 and AcrIF23 suppress Cas2/3 recruitment or enzymatic activity through direct binding" is misleading, since it implies that these are known to be the only two proteins that act though this mechanism.

I suggest rephrasing to "Notably, only AcrIF3 and AcrIF23 have been shown to suppress Cas2/3 recruitment or enzymatic activity through direct binding".

Our responses:

We sincerely appreciate the reviewer's insightful comment. As correctly noted, we have rigorously revised the text to ensure precise terminology and enhanced scientific rigor, with all modifications implemented in the relevant sections (Lines 79-81).

2. Results, line 157-168: I do not agree with the authors that the AcrIF19 beta-2 beta-3 "mimics ssDNA". I do not dispute that AcrIF19 occludes the ssDNA binding site on Cas2/3, and uses both negatively charged and aromatic residues to do so. But, the authors would need to demonstrate that these residues form structurally equivalent interactions with Cas2/3 as are observed in ssDNA complexes to support this strong claim. I suggest the authors rephrase their claims to simply state that AcrIF19 occludes the ssDNA binding site and thereby prevents ssDNA binding by Cas2/3.

Our responses:

We sincerely thank the reviewer for insightful comments on the mechanistic interpretation of AcrIF19's β_2–β_3 loop. We agree our structural and biochemical evidence supports spatial occlusion of the ssDNA-binding channel (Channel 1) instead of ssDNA functional mimicry. As suggested, we have revised all relevant sections to remove claims of "molecular mimicry" or "ssDNA mimicry", revised the section title to AcrIF19's β_2–β_3 Loop Induces Steric Blockade of Cas2/3 ssDNA Channel, and unambiguously clarified that: "AcrIF19 sterically occludes Channel 1 via its β_2–β_3 loop, thereby competitively inhibiting ssDNA binding."

3. Lines 175-176: The notation of amino acids interacting between AcrIF19 and Cas2/3 is confusing. Can the authors provide an indication here of which protein they are referring to in each case (as in the following sentences, where this is noted)?

Our responses:

We sincerely apologize for the ambiguity in the description of this section. We have made revisions at the corresponding position (Lines 170-174).

4. Results section: how many examples of AcrIF19 homologs are available? It would be helpful to show that the residues the authors discuss and mutate (in the beta-2 beta-3 loop and the C-terminus) are highly conserved in this family of proteins.

Our responses:

We appreciate the reviewer's suggestion. We identified 2 additional homologs (WP_121304167.1 and WP_121268704.1 from Pectobacterium parmentieri) via BLAST, totaling 3 sequences analyzed. The β_2-β_3 loop (E19, Y21–Y29) and C-terminus (Y89–I93), which are key for Cas2/3 inhibition, are conserved across all homologs. Our prior mutagenesis (E19R, E23R, E27R and ΔTail) showed these residues are essential for activity, supporting their functional importance. Thank you for improving the study's rigor.

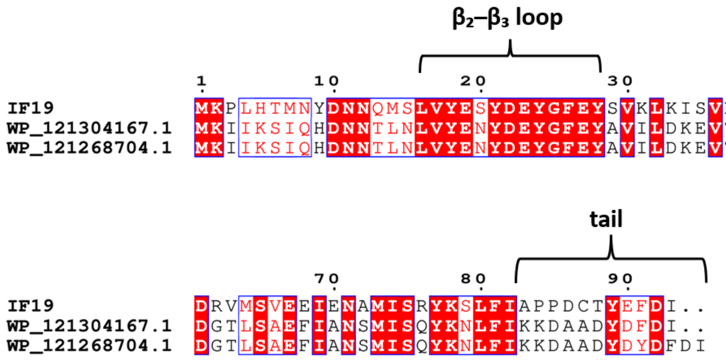

Response Fig. 1 Homology analysis of AcrIF19. The β_2–β_3 loop sequence of AcrIF19 is highly conserved, whereas its C-terminus sequence is partially conserved

5. Lines 281-296: The discussion of how the Cas2/3 "helix-like loop" moves in response to AcrIF19 binding, and the accompanying figure, is confusing. How do the authors know that this loop changes position upon AcrIF19 binding, since they are comparing the structure to an uninhibited structure of a completely different Cas2/3 protein? From Figure 6a, it looks like the position of the helix-like loop in the ICP1 Cas2/3 protein would be impossible for the PatCas2/3 helix-like loop, since part of the folded PatCas2/3 protein overlaps this loop's position in ICP1? Please explain and show this more clearly.

Our responses:

We sincerely appreciate the reviewer's insightful comments and apologize for the ambiguity in the original structural comparison and mechanism description. Our initial inference that "AcrIF19 induces conformational change of Cas2/3's helix-like loop" relied on indirect comparisons: the activated state of homologous ICP1 Cas2/3 (R-loop-bound, upward-swinging loop) and predicted apo/ssDNA-bound PatCas2/3 structures (both open-loop). As the reviewer noted, this was limited by cross-species structural differences and lack of experimentally determined apo PatCas2/3 data. To address this, we resolved the cryo-EM structure of apo PatCas2/3. Key finding: apo PatCas2/3 naturally adopts a closed auto-inhibitory state (downward-swinging loop blocking HD active center). Thus, Cas2/3 activation requires R-loop/ssDNA-induced upward loop swing to relieve auto-inhibition, while AcrIF19 functions by stabilizing this closed state (blocking activation) rather than inducing conformational change. The conflicting loop positions between ICP1 and PatCas2/3 reflect distinct functional states (activated vs. native auto-inhibited) rather than species-specific irreconcilable differences. We have optimized Figure 6 with structural superposition, labeled functional states to clarify this. Due to the revision of AcrIF19's mechanism, we have made extensive revisions to this chapter to elaborate on the molecular mechanism by which AcrIF19 inhibits the activation process of Cas2/3. We have fully reconstructed the relevant discussion, revised the mechanism description, and integrated supplementary data/optimized figures into the revised manuscript.

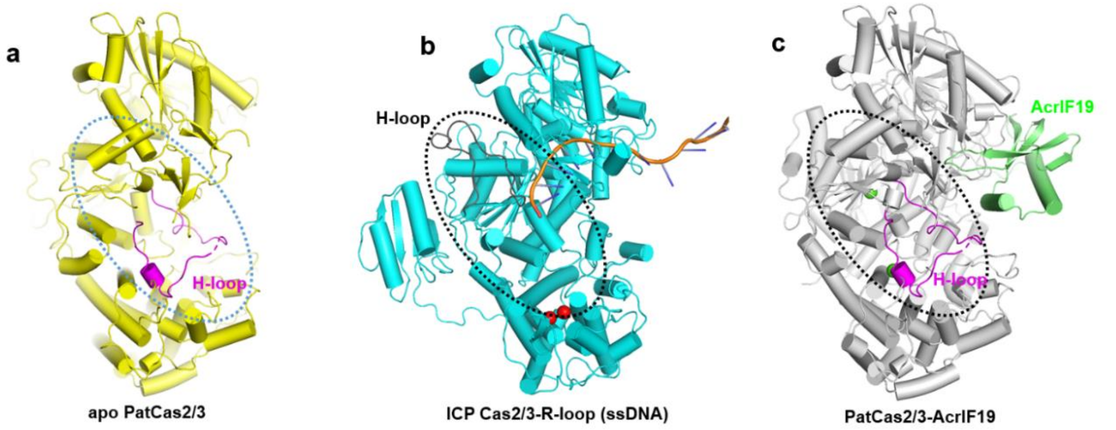

Response Fig. 2 AcrIF19 inhibits Cas2/3 activation. (a) Apo PatCas2/3 (yellow cartoon), helix-like loop in burgundy (swinging downward); (b) Cas2/3 and R-loop-ssDNA from Csy-dsDNA-Cas1-Cas2/3 (8K22), helix-like loop in black (swinging upward); (c) PatCas2/3-AcrIF19 complex, helix-like loop in burgundy (swinging downward).

6. Lines 298-306: The comparison of inhibition mechanisms between AcrIF3 and AcrIF19 suffers from the same problem as above. The authors do not have an unliganded structure of PatCas2/3, so they are comparing to a homologous protein. How do the authors know that the structural differences they highlight are due to AcrIF3/19 binding, not simply different between the two Cas2/3 proteins being compared?

Our responses:
We sincerely appreciate the reviewer for raising this critical question. In light of the reviewer's valuable comments, we have successfully determined the cryo-EM structure of monomeric PatCas2/3. Structural analysis demonstrates that the helix-like loop motif of monomeric PatCas2/3 inherently adopts an inward-swung conformation, which directly occludes the active site of the HD domain. This structural evidence confirms that AcrIF19 does not induce conformational changes in the helix-like loop motif of PatCas2/3. Meanwhile, we fully agree with the reviewer's concern that direct comparison of the conformational differences of Cas2/3 between the cross-species complexes (PatCas2/3-AcrIF19 and PaeCas2/3-AcrIF3), especially those involving minor conformational changes, makes it impossible to distinguish whether these differences arise from Acr binding or are intrinsic to the Cas2/3 orthologs from different species. Therefore, we have removed this comparative section from the original manuscript and will instead present a more rigorous discussion on the mechanistic differences between the two Acr proteins in the Discussion section, by integrating the structural data of monomeric PatCas2/3 obtained in this study.

7. Please add PAE diagrams and pTM/ipTM scores for all AlphaFold predictions.

Our responses:
We thank the reviewer for this important suggestion to improve the transparency and evaluation of our AlphaFold predictions. We have added the PAE (Predicted Aligned Error) diagrams and the corresponding pTM/ipTM scores for all AlphaFold-predicted structures to the revised manuscript, and these data are presented in the relevant supplementary figures/extended data with clear annotations in the figure legends to allow readers to assess the reliability of the AlphaFold models.

8. Lines 322-324: Please explicitly note here that the "structural analysis" being discussed is based on a predicted structure, not an experimental structure. How confident is this structure?

Our responses:

The reviewer raised a key critical question. We have explicitly indicated that the AcrIF23-Cas2/3 analysis utilizes an AlphaFold3-predicted complex (now clearly stated in Lines 327-331). While the model shows moderate confidence (ipTM=0.65, PTM=0.83), its biological relevance is supported by published biochemical validation: Ren et al. (2022, JBC. 298: 102124) demonstrated that alanine substitutions at E25/T26 severely impair AcrIF23's suppression activity, confirming these residues as essential interaction sites[1]. Our functional data further corroborate this mechanism, showing that AcrIF23 exclusively inhibits ssDNA cleavage without impeding recruitment (Fig. 7d, g); its binding at this site well accounts for its selective interference mechanism. Collectively, we hold that this predictive modeling combined with solid experimental evidence confers high confidence in our core mechanistic interpretation.

Figures:

9. Figure 1a/b: the AcrIF19 concentrations used in these assays are not noted in either the figure itself or the figure legend. In the current figure, the text "(uM)" above each gel is not informative, because there are no numbers for AcrIF19 concentration (only a triangle indicating increasing concentrations). The same problem exists in Extended Data Figure 1a.

Our responses:

We thank the reviewer for pointing out this oversight, which has now been rectified in the revised manuscript. We have added the exact AcrIF19 concentrations (in µM) corresponding to the gradient to both the panels and legends of Figure 1a/b and Extended Data Figure 1a, replacing the uninformative "(µM)" text with specific numerical values that align with the increasing concentration triangles. All concentration annotations are presented uniformly across these figures to ensure clarity and reproducibility of the experimental conditions for readers.

10. Figure 2a: It would be helpful for a reader if the authors included a domain diagram (from N to C terminus) of the different domains of Cas2/3, and the colors of each domain as used in this panel. I see the authors included exactly this panel as Extended Data Figure 2e - can this be moved to the main text figures?

Our responses:

We thank the reviewer for this constructive suggestion. We have moved the domain diagram of Cas2/3 (from N- to C-terminus) with color-coded annotation of each domain from Extended Data Figure 2e to the main text as an updated Figure 2a (now incorporating the domain diagram and the original content of Figure 2a for direct reader reference). All color designations for the Cas2/3 domains in this panel are kept consistent with those used throughout the manuscript to ensure visual coherence.

11. Figure 2: It would be helpful if the authors labeled both the "loop" and "tail" elements in panels b and c, in addition to panel e.

Our responses:

We thank the reviewer for this valuable suggestion aimed at enhancing figure clarity. We have now added the labels for the "loop" and "tail" elements to Figures 2c and 2d, which are consistent with the labeling nomenclature and style adopted in Figure 2e. This modification ensures that key structural elements are uniformly annotated across the relevant panels, thereby improving the readability and interpretability of Figure 2 for readers.

12. Figure 5e: Please add labels on the figure, or list in the figure legend, the concentrations of each component used in this assay.

Our responses:

We thank the reviewer for this important suggestion. We have revised Figure 5e as requested: the exact concentrations of all components employed in this assay have been added to the figure legend with clear annotation, and key concentration information has also been labeled directly on the figure panel for intuitive reader reference.

Reviewer #2 (Remarks to the Author):

This is an interesting, comprehensive structural biology study that reports the cryo-EM structure of the Pectobacterium atrosepticum Cas2/3–AcrIF19 complex at 2.97 Å resolution. The work identifies a dual inhibitory mechanism in which AcrIF19 simultaneously blocks Cas2/3 recruitment and nuclease activity through ssDNA mimicry and allosteric conformational locking of the RecA1 loop. The manuscript is methodologically rigorous, mechanistically insightful, and broadly relevant to CRISPR–Acr research.

However, the presentation can be significantly improved by clarifying novelty relative to AcrIF3 and AcrIF23, tightening structural interpretations, and providing clearer biochemical validation of the proposed allosteric effect. While the concept of a dual mechanism is intriguing, more rigorous and direct evidence is required to clearly substantiate this claim.

Our responses:

We are sincerely grateful for your positive recognition of the scientific merit, methodological rigor and broad relevance of our study, as well as your valuable constructive guidance. In strict accordance with your requirements, we have completed the targeted experiments (cryo-EM structure determination of monomeric patCas2/3 and ssDNA cleavage kinetic assays), and revised the manuscript to address your key concerns: clarifying novelty relative to AcrIF3/AcrIF23, tightening structural interpretations, and substantiating dual inhibitory mechanism. Detailed explanations and supporting data are elaborated in the subsequent sections.

Major Comments:

1. The current text briefly notes that AcrIF3 blocks recruitment and AcrIF23 inhibits cleavage, but the discussion should compare binding interfaces and functional overlaps more directly.

Our responses:

Thank you for your constructive comment. We have revised the discussion of manuscript to directly compare the binding interfaces and functional overlaps of

AcrIF23, AcrIF3 and AcrIF19.

2. The term “dual-pathway inhibition” is conceptually strong. It is needed to clarify

Our responses:

We thank the reviewer for this insightful comment and for recognizing the conceptual merit of our original description of AcrIF19’s inhibitory mechanism. We fully concur with the critical need for a precise definition of the inhibitory mode; to eliminate potential terminological ambiguity, we have entirely eliminated the use of the term "dual-pathway inhibition" in the revised manuscript. Instead, we adopt the term dual-functional inhibition with cascade suppression effect, which more accurately captures AcrIF19’s authentic inhibitory mechanism. AcrIF19 employs a negatively charged β_2–β_3 loop to sterically occlude the non-target DNA strand entry channel, acting as a competitive inhibitor to disrupt Cas2/3 recruitment. Concurrently, this steric occlusion impedes ssDNA-mediated allosteric activation, which locks the critical helix-like loop motif in an inhibitory conformation and thereby abrogates DNA cleavage activity. All corresponding descriptions in the Results and Discussion sections have been revised accordingly to reflect this precise functional definition and ensure conceptual clarity.

3. Figure 6A and 6B: The authors claim that the helix-like loop in the RecA1 domain undergoes a 180° rotation upon binding of AcrIF19, based on structural comparison with Cas2/3 homologs from other species. However, it remains unclear whether this structural difference is species-specific or indeed induced by AcrIF19 binding. To support this claim, it would be important to determine the structure of apo PatCas2/3 (i.e., in the absence of AcrIF19). Since the current structure was determined by cryo-EM, it seems feasible to obtain an apo-state reconstruction by simply excluding AcrIF19 during sample preparation. Such verification appears necessary to support the claim of dual inhibition.

Our responses:

We sincerely appreciate the reviewer for raising this critical question. In light of your comments, we have solved the structure of monomeric PatCas2/3 and revised the conclusions of the manuscript. Unexpectedly, AcrIF19 does not induce the helix like

loop conformational changes; instead, it abrogates the activation of Cas2/3. Apo PatCas2/3 naturally adopts a closed auto-inhibitory state, with its downward-swinging loop occluding the HD active center (**Response Fig. 2a**). Cas2/3 activation thus requires an upward conformational swing of this loop induced by R-loop or ssDNA to alleviate auto-inhibition, whereas AcrIF19 exerts its inhibitory effect by stabilizing this closed state—thereby blocking activation—rather than inducing de novo conformational changes. The divergent loop conformations between ICP1 and PatCas2/3 reflect distinct functional states (activated versus native auto-inhibited) rather than irreconcilable species-specific differences.

Again, we wish to thank the reviewer for his constructive comments and suggestions, which have helped us to improve our manuscript greatly.

4. The authors should provide quantitative ssDNA cleavage kinetics showing that AcrIF19 disrupts catalysis beyond steric blocking

Our responses:
We acknowledge the critical importance of the reviewer's point. In accordance with this guidance, we conducted detailed enzymatic kinetic analyses of Cas2/3 nuclease activity on FAM and BHQ Tag labeled ssDNA substrates[2], both in the absence and presence of AcrIF19. The results demonstrate that AcrIF19 reduces the maximum reaction velocity (V_{max}) while increasing the Michaelis constant (K_m). This kinetic profile establishes AcrIF19 as a mixed-type inhibitor; its suppression mechanism involves not only competitive steric occlusion but also the impediment of Cas2/3's conformational transition to its activated state.

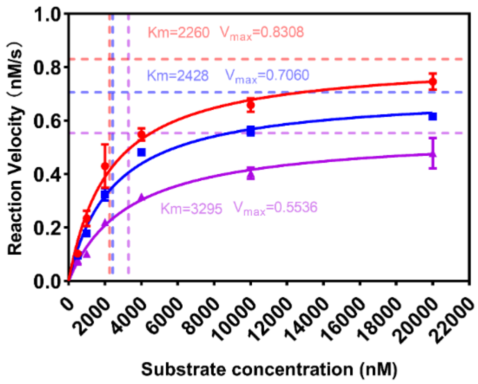

Response Fig. 3 Michaelis-Menten kinetic curves for Cas2/3 ssDNA cleavage activity in the absence (red) or presence of 0.045 μM (blue) or 0.09 μM (purple) AcrIF19. Reaction velocity (nM/s)

is plotted against substrate concentration (nM). Fitted kinetic parameters (K_m and Vmax) are labeled for each condition; data are from three independent experiments (error bars represent SD).

5. Cryo-EM map quality for the remodeled RecA1 loop (residues 488–523) is described as “partially unresolved”; representative density maps should be included to verify model confidence.

Our responses:

We thank the reviewer for this insightful comment. In response, we present the cryo-EM density map of the PatCas2/3-AcrIF19 complex with focused analysis on the RecA1 loop (residues 488–523). Structural examination reveals well-defined electron density for residues 488-501 and 511-523 of the helical-like loop. Crucially, the density at positions 510-523 demonstrates a downward conformational shift positioning this motif within the catalytic core of the HD nuclease domain. While interpretable density is absent for residues 502-510 – indicating inherent flexibility in this segment – the overall density map confirms the helical-like loop occupies the enzymatic active site region.

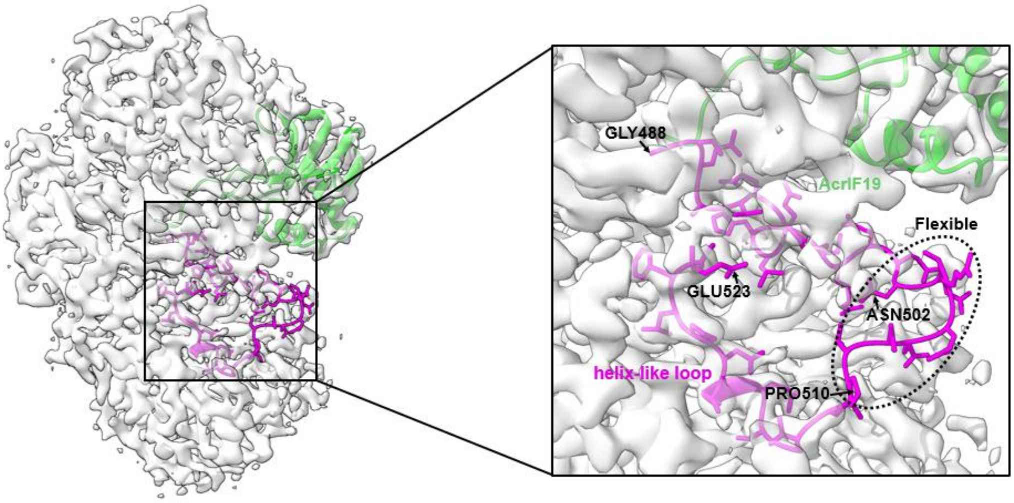

Response Fig. 4 Cryo-EM density map (contoured at 0.33σ, local resolution 2.7 Å) of the Cas2/3-AcrIF19 complex depicts the burgundy helical-like loop (residues 488-523) in RecA1 domain and green AcrIF19 inhibitor embedded within silver overall density.

Minor Comments

1. β_2–β_3 sometimes written as β2–β3

2. 2mg/mL or 2mgmL-1

3. Reference21 is not related to Acr. Please check it again.

Our responses:

We sincerely apologize for the writing errors identified in the manuscript draft. We have comprehensively addressed all issues raised by the reviewers made the following revisions to the manuscript:

(1) β_2–β_3 and β2–β3 have been uniformly standardized to β_2–β_3;

(2) mg/mL and $mgmL^{-1}$ formats have been uniformly standardized to mg/mL;

(3) References have been corrected to the appropriate citations.

Reviewer #3 (Remarks to the Author):

This study investigates the inhibitory mechanism of the previously discovered anti-CRISPR protein AcrIF19, which inhibits the type I-F CRISPR-Cas system of Pectobacterium atrosepticum. This system is composed of a multi-subunit complex that binds targeted dsDNA and recruits the helicase-nuclease Cas2/3 for target degradation. The authors use in vitro DNA cleavage assays and Cas2/3 recruitment assays, and cryoEM analysis to uncover the structural basis for inhibition by IF19. They find that IF19 occupies two important sites within Cas2/3. First, IF19 fits snugly into the same channel that Cas2/3 uses to bind its non-target ssDNA substrate, and the authors identify a loop important for this role. Secondly, the C-terminal tail of IF19 occupies and inhibits the HD nuclease active site of Cas2/3.

Overall the findings are interesting and the experiments well-executed. However, the authors do overstate their conclusions at times, with more experimental data required to substantiate their claims.

Our responses:

We sincerely appreciate the reviewer's recognition of our findings' novelty, fully concurring that some conclusions were overstated and required additional experimental evidence to substantiate core claims; we have refined the wording of conclusions and discussions for greater prudence by removing over-interpretation, and supplemented targeted experimental data to consolidate key arguments, with gratitude for the

reviewer’s comments that have enhanced the manuscript’s overall rigor.

1. The authors repeatedly frame AcrIF19 as having dual mechanisms of inhibition – it both blocks the ssDNA entry channel and blocks the HD active site responsible for ssDNA cleavage. They nicely show the counterexamples of AcrIF3 (which inhibits Cas2/3 recruitment but not ssDNA cleavage) and AcrIF23 (which does the opposite). However, though the authors comment on it in Lines 259-262, the separability of these dual mechanisms is not experimentally demonstrated for AcrIF19, in fact, some experimental conclusions seem to argue against it. If each mechanism were sufficient for Cas2/3 inhibition, then mutations abolishing each of the two mechanisms individually should not abolish inhibitory activity, as the other activity could compensate. Furthermore, the effects of these individual mutations might be expected to influence only the impacted arm of the pathway, i.e. Cas2/3 recruitment or ssDNA cleavage. Instead, the authors demonstrate that both (i) charge reversal mutations in the loop occupying the ssDNA entry channel, and (ii) deletion of the C-terminal tail that inhibits the HD active site, abolish both Cas2/3 recruitment and ssDNA cleavage. The total loss of activity by both sets of mutations argues for a unified inhibition mechanism rather than a dual one. It is perhaps most easily explained by a model in which both the loop and the tail are required for stable binding of AcrIF19 to Cas2/3, and interaction experiments along these lines could be informative. Until the authors demonstrate some separability of these two activities, their conclusion of a dual inhibitory mechanism is unsupported.

Our responses:

We sincerely appreciate the reviewer's insightful critique regarding the mechanistic characterization of AcrIF19. We concur that the initial "dual pathway" terminology inadequately describes its inhibitory mechanism. Our newly resolved cryo-EM structure of monomeric PatCas2/3 (Fig. 6a-d) reveals that Cas2/3 adopts an autoinhibited basal conformation requiring R-loop/ssDNA binding for activation. AcrIF19 employs its negatively charged β_2–β_3 loop to sterically occlude the non-target DNA strand entry channel, competitively inhibiting Cas2/3 recruitment. This occlusion concurrently prevents ssDNA-mediated allosteric activation, locking the catalytic helical-loop motif in an inhibitory conformation that abolishes DNA cleavage. Supporting this integrated mechanism, enzyme kinetics establish AcrIF19 as a mixed-

type inhibitor (Response Fig. 3). Functional validation via Microscale Thermophoresis (MST) demonstrates hierarchical binding affinities: Cas2/3 exhibits strongest binding to target ssDNA (K_d = 101.39 ± 48.6 nM), while wild-type AcrIF19 shows intermediate affinity (K_d = 284.58 ± 155.81 nM). Strikingly, charge-reversal mutants (E19R/E23R/E27R) and C-terminal truncation (ΔTail) exhibit 3.77- to 15.6-fold and 101.70-fold affinity reductions respectively, confirming that both structural domains are essential for stable complex formation (Response Fig. 5). Collectively, these data demonstrate AcrIF19 exerts cascade dual-function inhibition: (1) competitive blockade of DNA recruitment, and (2) disruption of allosteric activation. Consequently, we agree that 'dual pathway' was misleading as it implies two separable inhibitory routes. Our data demonstrate an integrated mechanism: AcrIF19's C-terminal tail anchors to Cas2/3, enabling its β_2-β_3 loop to competitively block DNA binding. This steric occlusion concurrently prevents allosteric activation via ssDNA, as evidenced by: we have revised the mechanistic descriptor to "cascade dual-function inhibition" in the updated manuscript.

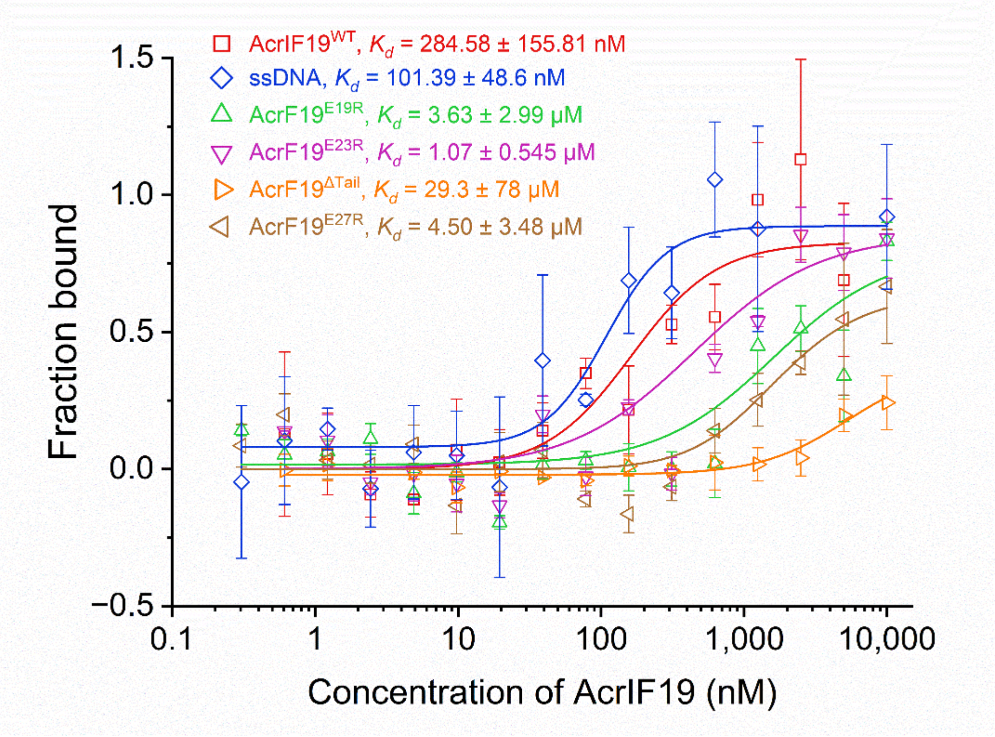

Response Fig. 5 Interaction between AcrIF19 and Cas2/3 is essential for its inhibition activity. MST assay of the binding between the purified Cas2/3 and AcrIF19 or AcrIF19 mutants. The binding curves are displayed. Shown are mean values ± s.d.; n = 3. Individual data points are also shown.

2. The authors conclude that the loop of AcrIF19 is a "structural mimic" of ssDNA. This case is much less supported than other established examples of structural mimicry,

where a protein exhibits the same fold and charge distribution as another macromolecule, e.g. PAM-containing DNA mimicry by AcrIIA4. In the case of the AcrIF19 loop, the authors argue that because the loop contains negatively charged residues and occupies the ssDNA entry channel, it is a mimic of ssDNA. By this logic, any drug that occupies an enzyme's active site could also be considered a mimic of the enzyme's substrate. I recommend that the authors eliminate the term "mimic" and instead refer to AcrIF19 as a competitive inhibitor of ssDNA binding. Further data to support this conclusion could also come from testing whether positively charged residues contacted by AcrIF19 in the ssDNA entry channel are required for DNA binding and cleavage by Cas2/3.

Our responses:

We sincerely thank the reviewer for highlighting the overinterpretation of 'structural mimicry'. We fully accept this critique and have eliminated all such terminology. AcrIF19 is now consistently described as a competitive inhibitor that sterically occludes the ssDNA entry channel via its β_2-β_3 loop. This revised interpretation is robustly supported by existing data: (1) competitive inhibition kinetics showing increased K_m and decreased V_{max} (Response Fig. 3), (2) cryo-EM visualization of loop occupancy in Channel 1 (Fig. 3b), and (3) dose-dependent disruption of ssDNA binding (Fig. 5e). Collectively, these findings confirm competitive inhibition as the operative mechanism.

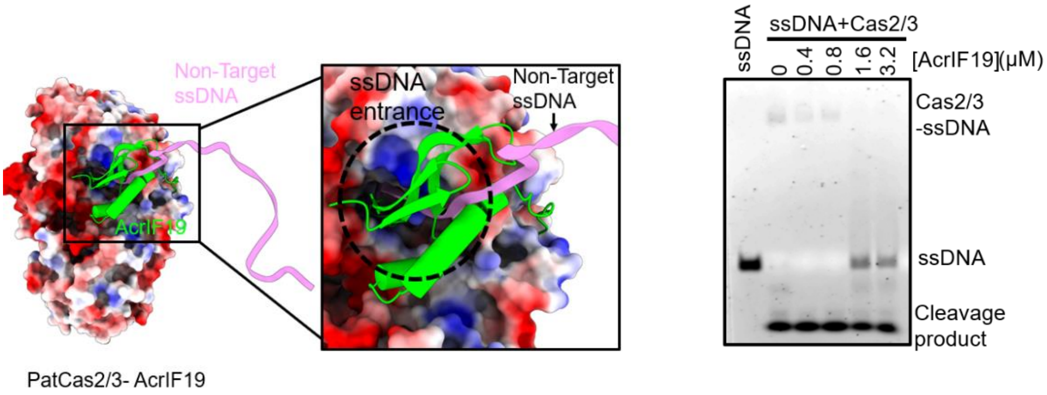

Response Fig. 6 Competitive binding between AcrIF19 and ssDNA for Cas2/3. (Left) Structural overlap of AcrIF19 (green) and ssDNA (pink) binding sites on Cas2/3, demonstrating spatial competition at the Cas2/3 interface (Fig. 3b). (Right) Concentration-dependent disruption of Cas2/3-ssDNA complexes by AcrIF19 (Fig. 5e), confirming direct competitive inhibition through binding site occlusion.

3. In the abstract, and throughout the manuscript, the authors claim that AcrIF19

represents the first Acr with a “dual-pathway” inhibitory mechanism. This is not true, as it was previously shown that AcrIIA1 inhibits Cas9 via direct binding to the HNH domain and via induction of Cas9 degradation, and that these are separable activities (see Osuna et al. 2020 Cell Host & Microbe). This claim of priority is unnecessary and needs to be removed.

Our responses:

We thank the reviewer for pointing out this critical oversight and for the valuable reference. We fully agree with the comment that the priority claim for the "dual-pathway" inhibitory mechanism is inappropriate and unnecessary, and we have completely removed all such priority-related statements (including the claim that AcrIF19 represents the first Acr with this inhibitory mode) from the abstract and all relevant sections throughout the revised manuscript. Furthermore, to eliminate terminological ambiguity, we have discontinued "dual-pathway inhibition" throughout the revised manuscript. AcrIF19's inhibitory mechanism is now consistently defined as cascade dual-function inhibition of Cas2/3 – concurrently disrupting ssDNA substrate recruitment and HD nuclease catalytic cleavage through structurally coordinated mechanisms.

We sincerely apologize for this error in the original manuscript and appreciate the reviewer’s guidance in improving the accuracy and rigor of our work.

4. The introduction is longwinded (paragraph 1 especially), with superfluous discussion about all the different CRISPR classes and types, adaptation and crRNA processing, which have been covered extensively elsewhere and are not really the focus of this manuscript. With the space made by this trimming, the authors should include more information regarding Cas2/3’s ssDNase activity, which does feature centrally in the manuscript. Please explain the role of the ssDNase activity and how this results in degradation of dsDNA (Line 68).

Our responses:

We appreciate the reviewer's suggestion and have streamlined the introduction, particularly the first paragraph, by removing peripheral discussions on CRISPR classification, adaptation mechanisms, and crRNA processing while preserving core

concepts to ensure conceptual continuity. Utilizing the space made available by these revisions, we have: Elaborated on Cas2/3's ssDNase activity and its functional significance, (ii) Clarified the mechanistic basis of dsDNA degradation, with detailed explanations now incorporated in lines 56-63 of the revised manuscript. The introduction now better focuses on the manuscript's core.

5. Fig. 1 and Extended Data Fig. 1: Legends say AcrIF19 was added with the indicated concentrations. The concentrations are not indicated, there is only a triangle. Also relevant for Fig. 3d, 5e,7d-i. In all cases, please indicate concentrations or molar ratios of all Cas proteins / Acrs / substrates.

Our responses:
We sincerely appreciate your pointing out the omission in the figure legends. As requested, we have revised all relevant figures (Fig.1, Extended Data Fig.1, Fig.3d, 5e, and 7d-i).

6. Fig. 1c: Please show the tract of Cas2/3 alone (with no Acr) as this is the relevant control trace. The Csy+AcrIF19 trace shown is uninformative.

Our responses:
We highly appreciate the reviewer's critical and constructive suggestion. We have added the relevant control trace of Cas2/3 alone (without Acr) in the revised Fig. 1c as required. Meanwhile, we have retained the Csy+AcrIF19 trace because we want to confirm that AcrIF19 inhibits the system by targeting Cas2/3 rather than the Csy complex (Line112-116).

7. I do not think it is required for this manuscript, but in general for future DNA cleavage assays, a non-targeted control substrate would be helpful.

Our responses:
We appreciate the reviewer's suggestion. We agree that a non-targeted control substrate would benefit DNA cleavage assays. We will incorporate this control in our future DNA cleavage assays to improve experimental rigor.

8. Fig. 2b-c: Please label the Loop and Tail of AcrIF19.

Our responses:

We appreciate the reviewer's suggestion. We have clearly labeled the Loop and Tail of AcrIF19 in the revised Fig. 2b-c (now adjusted to Fig. 2c-d).

References

1. Ren, J. *et al.* Structural and mechanistic insights into the inhibition of type I-F CRISPR-Cas system by anti-CRISPR protein AcrIF23. *J Biol Chem* **298**, 102124 (2022).

2. Ramachandran, A. & Santiago, J.G. CRISPR Enzyme Kinetics for Molecular Diagnostics. *Anal Chem* **93**, 7456-7464 (2021).

REVIEWER COMMENTS

Reviewer #1 (Remarks to the Author):

The authors have done a good job addressing all reviewer questions. I support publication.

Reviewer #2 (Remarks to the Author):

The authors have resolved the apo structure of Cas2/3 and proposed a new mechanism based on it, adequately addressing all of my previous comments. My concerns have been fully resolved, and I recommend the manuscript for acceptance.

Reviewer #3 (Remarks to the Author):

The revised manuscript is much improved, with additional experimental data and appropriately toned down conclusions. I support publication and congratulate the authors on an excellent study.

We thank the reviewers for their positive comments and for taking the time to review our manuscript.

Reviewer #4 (Remarks to the Author):

The paper studies AcrIF19, an anti-CRISPR protein that inhibits the type I-F CRISPR-Cas system in Pectobacterium atrosepticum by targeting Cas2/3, the nuclease-helicase that carries out DNA degradation. The main claim is that AcrIF19 is unusual because it blocks both recruitment of Cas2/3 to the target DNA complex and its nuclease activity once bound. The authors support this with cryo-EM, mutagenesis, binding assays, cleavage assays, and comparisons with other anti-CRISPRs.

The main result of the paper is that AcrIF19 binds directly to Cas2/3 rather than the Csy surveillance complex. Structurally, AcrIF19 uses two sections of the protein:

1.- A beta2-beta3 loop that plugs the ssDNA entry channel on Cas2/3, blocking access of the non-target DNA strand and impairing Cas2/3 recruitment

2.- A C-terminal tail that acts as an anchor to stabilize binding to Cas2/3.
The paper argues that this binding also keeps a RecA1 “helix-like loop” in a position that blocks the catalytic channel near the HD nuclease site, preventing DNA cleavage. In the revised manuscript, the authors frame this as an integrated cascade dual-function inhibition mechanism rather than two

independent pathways.

We appreciate the reviewer's high evaluation of our work and constructive suggestions. We have addressed the comments listed below and have revised the manuscript accordingly. Taken together, we believe that these revisions according to the reviewer's comments have significantly improved our manuscript.

After analyzing the maps and models and the claims in the paper, I think the authors should revised D22 in the beta2-beta3 loop, as it seems the side chain is out of teh density. The authors can adjust the residue but I think this may change some of teh contacts that they have described in the corresponding figure. I think the main conclusion is still fine.

Our response:

We are grateful for the reviewer's critical suggestion. We have revised D22 in the beta2-beta3 loop and also updated the corresponding Fig. 3e. As the reviewer noted, the main conclusion is still fine, and we only made some minor revisions to the corresponding text in the revised manuscript.

However, the authors have what I think is 1-2 cations bound between His217, His 216, His361, D124, D324. I think this is the active center of the HD domain. Previous structures have modeled the density as Mn, but could be Ni which was used in the purification. I think the authors should check, whether the isolated Cas2/3 nuclease activity its OK. In a case of bad luck it could be that the lack of cleavage that they observed is due to an inhibition of the nuclease by a cation that binds and does not favor catalysis.

For HD nucleases, the effect of Ni is enzyme-specific some HD-domain enzymes are active with that cation and some show reduced activity compared with Mn and Fe. In some cases Ni can act as a poor substitute or become inhibitory depending on concentration and competition with the native metal.

Our response:

The reviewer raised a very good point. We have revised the structure coordinates accordingly to include one and two Ni ions in the structures of AcrIF19-bound and apo Cas2/3 structure based on the map, respectively. Actually, the nuclease activity of the isolated PatCas2/3 is OK (lane 2 of Fig. 7f), suggesting that in the case of PatCas2/3, the Ni ion does not exhibit inhibitory roles. This is also the case of PaeCas2/3, which is also active as isolated from *E. coli* cells[1].

Again, we wish to thank the reviewer for her/his constructive comments and suggestions, which have helped us to improve our manuscript greatly.

Reference

1. Ren, J. *et al.* Structural and mechanistic insights into the inhibition of type I-F CRISPR-Cas system by anti-CRISPR protein AcrIF23. *J Biol Chem* **298**, 102124 (2022).
